# Frenkel-defected monolayer MoS_2_ catalysts for efficient hydrogen evolution

**DOI:** 10.1038/s41467-022-29929-7

**Published:** 2022-04-22

**Authors:** Jie Xu, Gonglei Shao, Xuan Tang, Fang Lv, Haiyan Xiang, Changfei Jing, Song Liu, Sheng Dai, Yanguang Li, Jun Luo, Zhen Zhou

**Affiliations:** 1 Institute of Functional Nano & Soft Materials (FUNSOM), Jiangsu Key Laboratory for Carbon-Based Functional Materials and Devices, Soochow University, 215123 Suzhou, China; 2 State Key Laboratory of Chemo/Biosensing and Chemometrics, College of Chemistry and Chemical Engineering, Hunan University, 410082 Changsha, Hunan China; 3 Engineering Research Center of Advanced Functional Material Manufacturing of Ministry of Education, School of Chemical Engineering, Zhengzhou University, 450001 Zhengzhou, China; 4 Feringa Nobel Prize Scientist Joint Research Centre, School of Chemistry and Molecular Engineering, East China University of Science & Technology, 200237 Shanghai, China; 5 School of Materials Science and Engineering, Tianjin Key Lab of Photoelectric Materials & Devices, Tianjin University of Technology, 300384 Tianjin, China

**Keywords:** Characterization and analytical techniques, Electrocatalysis, Nanoscale materials

## Abstract

Defect engineering is an effective strategy to improve the activity of two-dimensional molybdenum disulfide base planes toward electrocatalytic hydrogen evolution reaction. Here, we report a Frenkel-defected monolayer MoS_2_ catalyst, in which a fraction of Mo atoms in MoS_2_ spontaneously leave their places in the lattice, creating vacancies and becoming interstitials by lodging in nearby locations. Unique charge distributions are introduced in the MoS_2_ surface planes, and those interstitial Mo atoms are more conducive to H adsorption, thus greatly promoting the HER activity of monolayer MoS_2_ base planes. At the current density of 10 mA cm^−2^, the optimal Frenkel-defected monolayer MoS_2_ exhibits a lower overpotential (164 mV) than either pristine monolayer MoS_2_ surface plane (358 mV) or Pt-single-atom doped MoS_2_ (211 mV). This work provides insights into the structure-property relationship of point-defected MoS_2_ and highlights the advantages of Frenkel defects in tuning the catalytic performance of MoS_2_ materials.

## Abstract (web summary)

While material defect sites are active for chemical reactions, it is important to understand how different defect types impact reactivity. Here, authors prepare Frenkel-defected MoS_2_ monolayers and demonstrate improved performances for H_2_ evolution electrocatalysis than pristine or doped MoS_2_.

## Introduction

The world’s growing energy demand based on the finite fossil fuel resources combined with the documented anthropogenic effects on the climate have ignited the research passion for renewable energy technologies. Currently, one area of emphasis is materials and systems for electrocatalytic water splitting (particularly hydrogen evolution reaction, HER) to produce hydrogen as an alternative energy resource due to its high energy density and zero carbon emission in the combustion process^1,2^. An efficient and economical electrocatalyst is the key to achieving significant application for HER. Transition metal dichalcogenides (TMDs) are attracting wide attention due to their low cost and high HER activity. For example, two-dimensional (2D) TMD electrocatalysts are reported to possess enhanced HER performance than that of commercial benchmark Pt/C catalyst at high current densities^3,4^, showing great promise in industrial hydrogen production.

As a core member of the TMD material family, 2D molybdenum disulfide (MoS_2_) materials have been widely used in electrocatalytic HER^5,6^. It is generally considered that their active centers are the unsaturated coordinative atoms at the surface edges, however, the saturated coordinative atoms in the MoS_2_ surface base planes do not participate in the construction of catalytic active sites, losing the advantage of high specific surface areas of 2D MoS_2_ to some extent. Thus, several strategies, including defect engineering, surface modification, phase transition engineering, and stress–strain control^7–11^, have been proposed to increase the number of active sites on the MoS_2_ base planes and to optimize their HER performances.

Among them, defect engineering is an efficient measure, and particularly, point defects, e.g., substitution atoms, interstitial atoms, and atomic vacancies, may construct additional active sites in 2D MoS_2_ toward HER. For instance, the HER performance of 2D MoS_2_ was greatly improved via introduction of Pt single-atom dopants^7^ that modified the coordination environment and charge distribution on the MoS_2_ surface planes. In addition, Pd single-atom dopants on monolayer 1T-MoS_2_ were found to activate the planar inert atoms, accelerating the conversion rate to H_2_^12^. The synergistic effect between the point defects were also revealed to improve the HER performance of MoS_2_^13–15^. However, current defect engineering strategies on MoS_2_ are mostly based on heterogeneous atom doping^7,12,14–16^, as mentioned above, particularly noble metal doping, which may add the cost and complexity for synthesis approaches. Seeking a facile and economical defect engineering strategy on 2D electrochemical catalysts is of great importance. Meanwhile, the noble metal doping strategy shows limited space for electrocatalytic improvement only on account of the substitution atoms. Whether additional kinds of point defects may provide a desired structure-property relationship for 2D MoS_2_ is worthwhile exploring.

In this work, by referencing to the native point defects in the text books, we design and synthesize a Frenkel-defected monolayer MoS_2_ catalyst (denoted as FD-MoS_2_, including FD-MoS_2_-3, FD-MoS_2_-5 and FD-MoS_2_-15), in which the atomic configuration of the interstitial Mo atoms accompanying with the atomic vacancies is disclosed via aberration-corrected scanning transmission electron microscopy (AC-STEM) and additional spectroscopy characterizations. Micro-reactor electrochemical evaluation shows a significantly enhanced HER activity of the FD-MoS_2_-5 catalyst, of which the overpotential at the current density of 10 mA cm^−2^ is 164 mV, much lower than those of the reference Pt-single-atom doped monolayer MoS_2_ plane (211 mV) and the pristine monolayer MoS_2_ plane (358 mV). Density functional theory (DFT) calculation reveals the unique charge distribution and H adsorption sites introduced by the Frenkel defects in MoS_2_. These findings highlight the advantages of Frenkel defects in tuning the HER performance of 2D materials for outperforming Pt single-atom doped 2D catalysts.

## Results

### Synthesis and characterization

**Fig. 1:**
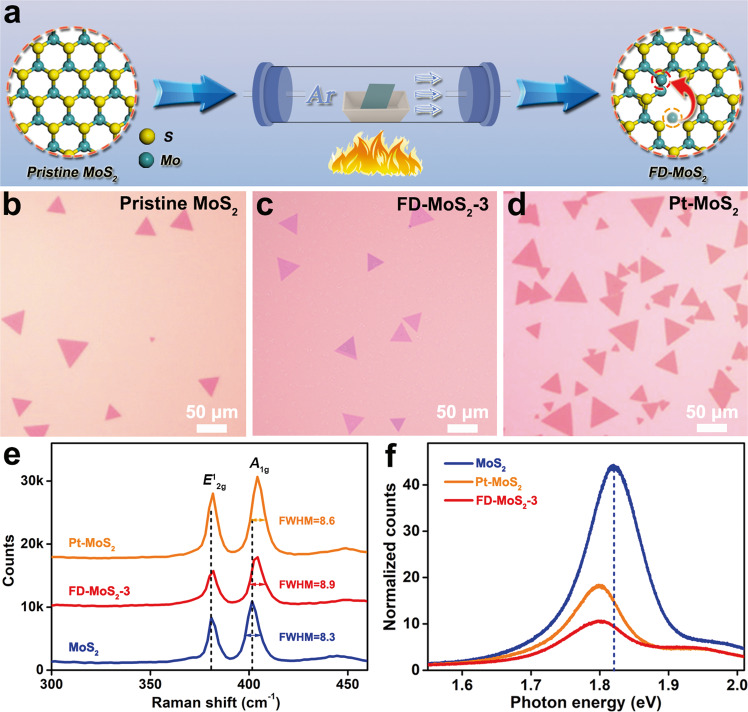
Preparation and structural characterization of FD-MoS_2_. **a** Schematic synthetic procedure for FD-MoS_2_. **b**–**d** Typical optical images of monolayer intrinsic MoS_2_ (**b**), FD-MoS_2_-3 (**c**), and Pt-MoS_2_ (**d**). **e**, **f** Raman and normalized PL spectra of the three samples.

In our experiments, pristine monolayer MoS_2_ was initially prepared by a chemical vapor deposition (CVD) method. Then, the as-prepared MoS_2_ materials were annealed in Ar atmosphere at 400 °C for 3 min to obtain FD-MoS_2_-3, as illustrated in Fig. 1a. In addition, the Pt-single-atom doped monolayer MoS_2_ (denoted as Pt-MoS_2_) was also prepared via solution spin coating and CVD growth for comparison. Based on the optical microscopy observation, all the three kinds of monolayer MoS_2_–based materials showed regular triangle shapes (Fig. 1b–d). The thickness of FD-MoS_2_-3 was measured to be 0.76 nm via atomic force microscopy (AFM) in Supplementary Fig. 1, in accordance with the characteristic of monolayer MoS_2_ structures^16^.

Raman spectroscopy, photoluminescence (PL) spectroscopy, and X-ray photoelectron spectroscopy (XPS) were employed to investigate the structural information of the three MoS_2_ materials. As shown in the Raman spectra (Fig. 1e), the gap between *E*^1^_2g_ and *A*_1g_ vibration modes of the pristine MoS_2_ is ~20 cm^−1^, the same as that in the previous report^17^. Gauss fitting was used to analyze the full widths at half maximum (FWHM) of the Raman *A*_1g_ peak of the MoS_2_, Pt-MoS_2_, and FD-MoS_2_-3 samples, and the corresponding values were 8.3, 8.6, and 8.9 cm^−1^, respectively. The peak broadening of Pt-MoS_2_ and FD-MoS_2_-3 samples indicates that they have defective structures^18^. In addition, the Raman peaks of Pt-MoS_2_ are blue-shifted compared to pristine MoS_2_, which is related to point defects caused by foreign atom doping^19^. Figure 1f shows the normalized PL spectrum of pristine monolayer MoS_2_ (normalized to the baseline of PL), and its band gap is ~1.82 eV^20^. As for FD-MoS_2_-3 and Pt-MoS_2,_ the intensities of the main peaks are significantly suppressed, and both the main peaks show red-shifts. This is due to the change of electron concentration caused by the defect structure, which further confirms the existence of point defects in FD-MoS_2_-3 and Pt-MoS_2_^17,21^. We also independently collected the PL data from different regions of MoS_2_ and FD-MoS_2_-3 samples, demonstrating that the change of PL spectrum intensity of FD-MoS_2_-3 relative to MoS_2_ is reliable (Supplementary Fig. 2).

Moreover, the chemical states of these MoS_2_ materials were investigated by XPS, as shown in Supplementary Figs. 3 and 4. For the pristine MoS_2_, the peaks of Mo *3d*_*5/2*_ (at 229.7 eV) and *3d*_*3/2*_ (at 232.8 eV) are attributed to Mo^4+^, while the peaks of S *2p*_*3/2*_ (at 162.5 eV) and *2p*_*1/2*_ (at 163.7 eV) belong to S^2−^, demonstrating the high quality of as-synthesized pristine MoS_2_ over a large scale^17^. As for FD-MoS_2_-3 and Pt-MoS_2_, the peaks in the Mo *3d* and S *2p* regions show high binding-energy shifts, respectively. Such XPS peak shifts of Pt-MoS_2_ are consistent with a similar case in the literature, indicating the successful doping of Pt atoms in the MoS_2_ lattice^22^.

**Fig. 2:**
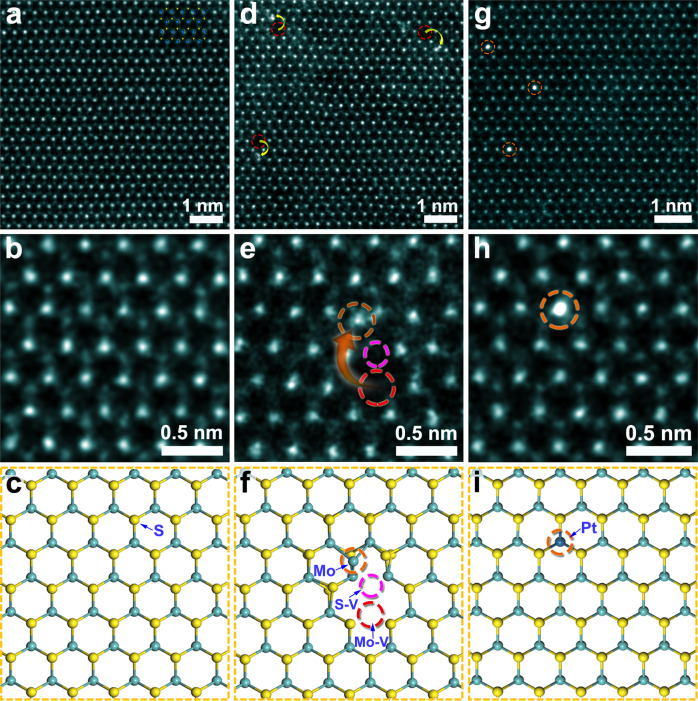
AC-STEM characterization of MoS_2_-based catalysts. Atomic-resolution HAADF-STEM images and corresponding atomic models of monolayer pristine MoS_2_ (**a**–**c**), FD-MoS_2_-3 (**d**–**f**), and Pt-MoS_2_ (**g**–**i**). S-V and Mo-V denote the S and the Mo vacancies, respectively.

AC-STEM was carried out to disclose the atomic configurations of these pristine and defected MoS_2_ materials. Figure 2a shows an overviewing high angle annular dark field (HAADF)-STEM image of the pristine MoS_2_, in which no obvious point defects (such as S or Mo vacancies) can be observed (more atomic-resolution HAADF-STEM images are provided in Supplementary Fig. 5). An enlarged HAADF-STEM image of the pristine MoS_2_ (along the <001> zone axis) is presented in Fig. 2b. Here, the high-contrast and low-contrast atoms correspond to Mo and S, respectively, as illustrated by the atomic projection model (Fig. 2c).

An overviewing HAADF-STEM image of FD-MoS_2_-3 is presented in Fig. 2d (more data are presented in Supplementary Fig. 6). The Frenkel defected sites are highlighted by the yellow arrows. It can be observed that a fraction of Mo atoms leave their own lattice sites, creating Mo vacancies and becoming interstitials by lodging in nearby locations, consistent with the definition of Frenkel defects^23^. More atomic-resolution HAADF-STEM images of different regions of FD-MoS_2_-3 are provided in Supplementary Fig. 7. Importantly, the intermediate state showing the formation of Frenkel defects was captured (Supplementary Fig. 7a, b). The enlarged image (Fig. 2e) illustrates the characteristics of Frenkel defects, and the corresponding atomic model is also presented (Fig. 2f). Accordingly, the S vacancies associated with the Mo Frenkel defects are also identified via elaborate intensity analysis on the Z-contrast HAADF-STEM images (Supplementary Fig. 8). Besides, the existence of S vacancies, but no Mo interstitial, is identified by AC-STEM (Supplementary Fig. 9) in monolayer MoS_2_ undergoing the Ar annealing for only 1 min. This evidence indicates that the S vacancies should be generated prior to the Mo migration in the formation of Frenkel defects.

In addition, the elapsed time of Ar annealing was extended to investigate its influence on the number of Frankel defects. For example, FD-MoS_2_-5 was obtained after 5-min Ar annealing and FD-MoS_2_-15 after 15-min. Compared with FD-MoS_2_-3, the Raman and PL spectra of FD-MoS_2_-5 and FD-MoS_2_-15 show more defect structures (Supplementary Fig. 10)^18,21^. More typical HAADF-STEM images also confirm the increased concentration of Frankel defects in FD-MoS_2_-5 (Supplementary Fig. 11). However, more holes were observed in addition to the Frenkel defects in FD-MoS_2_-15 (Supplementary Fig. 12), indicating that the monolayer MoS_2_ material was unstable and easy to decompose after Ar annealing for a rather long time.

For comparison, Fig. 2g shows the AC-STEM characterization of Pt-MoS_2_ (the raw data in Supplementary Fig. 13). Pt single atoms in different regions are clearly identified on Pt-MoS_2_ by HAADF-STEM imaging (Supplementary Fig. 14). Meanwhile, energy-dispersive X-ray spectroscopy (EDS) elemental maps (Supplementary Fig. 15) confirm the successful and homogeneous doping of Pt atoms. As shown in the HAADF-STEM image and the atomic structure model (Fig. 2h, i), the Pt atoms showing the highest Z-contrast in the image replace the Mo atoms in the MoS_2_ lattice, constructing a foreign-atom defect type which is different from that in FD-MoS_2_. Moreover, statistical analysis based on STEM observation, as reported in the previous work^6^, is utilized for the defect concentration measurement. As a result, the average concentration of Frenkel defects with the interstitial Mo atoms are measured to be 0.50% and 0.85% in FD-MoS_2_-3 (Supplementary Fig. 7) and FD-MoS_2_-5 (Supplementary Fig. 11), respectively, while the average concentration of Pt dopants in Pt-MoS_2_ is 0.80% (Supplementary Fig. 14).

DFT calculation was performed to investigate the formation process of Frenkel defects in monolayer MoS_2_. As revealed by the HAADF-STEM evidence (Supplementary Fig. 9), the Frenkel defects (for Mo atoms) were always observed along with the appearance of S vacancies that are easily induced during the Ar annealing process^24,25^. Therefore, it is reasonable that the formation of Frenkel defects in monolayer MoS_2_ is triggered by those S vacancies. Supplementary Figure 16a–d present the calculated reaction diagrams of monolayer MoS_2_ with different numbers of S vacancies (e.g., 3, 4, 5, and 6) for the formation of Frankel defects. As the increase of S vacancy number, the formation energy of Frenkel defects is significantly reduced (Supplementary Fig. 16e), and thermodynamic effects become dominant in the formation of Frenkel defects. Moreover, we calculated the formation energy of different numbers of S vacancies in pristine MoS_2_ at 673 K (Supplementary Fig. 16f). According to the result, it is feasible to generate S vacancies in the localized regions of MoS_2_ under our experimental conditions. The presence of S vacancies and the high annealing temperature will easily trigger the migration of Mo atoms to form stable Frenkel defects in MoS_2_.

### Electrocatalytic HER performances

**Fig. 3:**
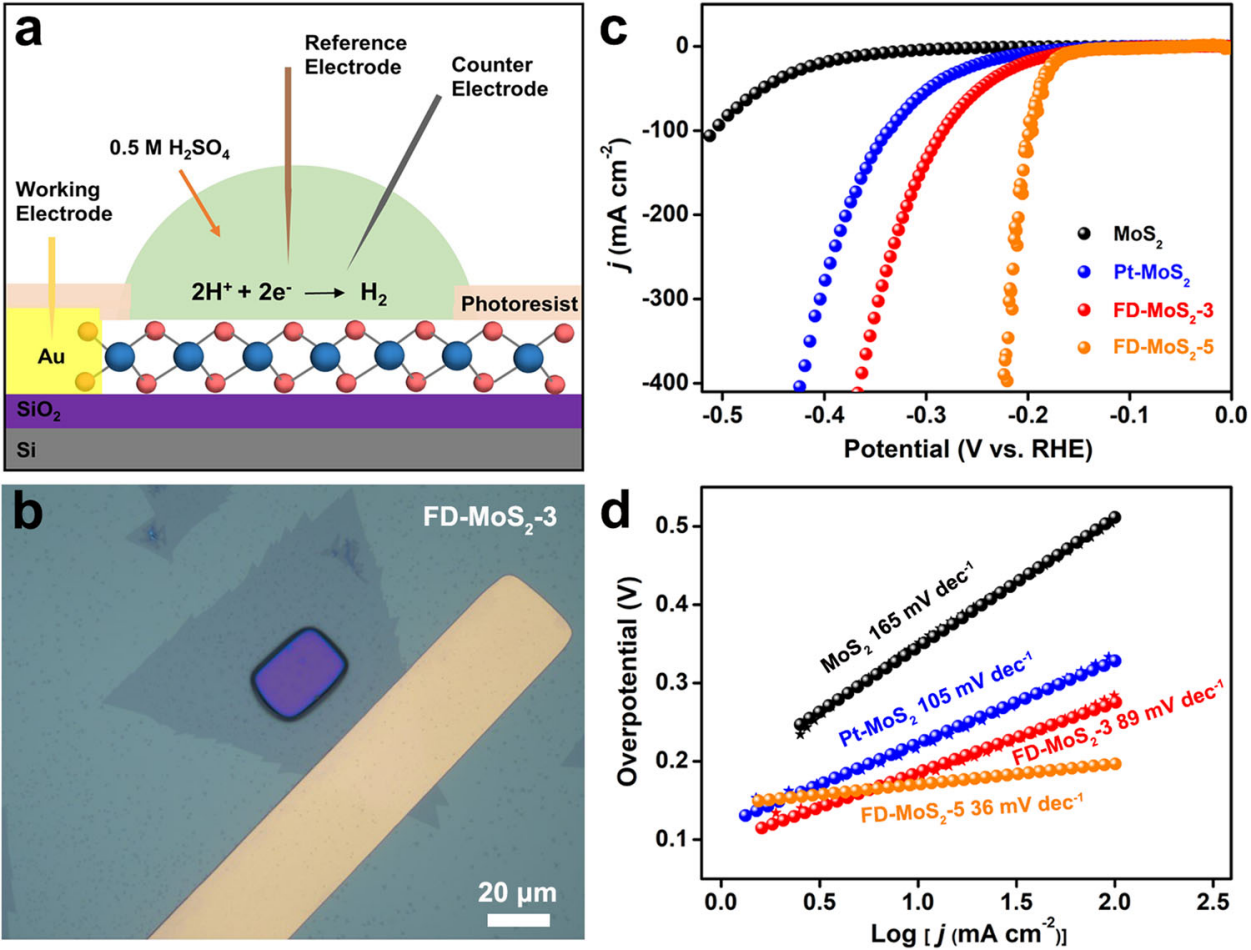
HER measurement and performance of the MoS_2_ catalysts by micro-electrochemical devices (micro-reactors). **a**, **b** Schematic and optical images of a micro-reactor. The micro-reactors are made by ultraviolet photolithography, and only the surface base plane of MoS_2_ is exposed in each device. The window area of each type of sample was 450 μm^2^ (pristine MoS_2_), 200 μm^2^ (Pt-MoS_2_), 600  μm^2^ (FD-MoS_2_-3), and 600 μm^2^ (FD-MoS_2_-5). In **b**, the yellow part is an In/Au electrode and the purplish red part is an exposed MoS_2_ base plane. The edges of the MoS_2_ samples are all covered by photoresist and did not participate in the HER. **c**, **d** Polarization curves and Tafel plots of monolayer pristine MoS_2_, Pt-MoS_2_, FD-MoS_2_-3, and FD-MoS_2_-5 in 0.5 M H_2_SO_4_. All current values are normalized by the exposed MoS_2_ surface areas.

Electrochemical test was then performed to evaluate the HER performances of the monolayer pristine MoS_2_, Pt-MoS_2_, FD-MoS_2_-3, and FD-MoS_2_-5 catalysts. For conventional electrochemical tests, catalysts are usually loaded on conductive substrates in the form of powders or films. However, for 2D catalysts, the activities of both edges and surface planes are included in this way, and the contributions of the point defects may not be differentiated from those of the unsaturated atoms at edges. To solve this problem, micro-electrochemical devices (Fig. 3a, b) are employed in our set-up^26–28^, and only the surface base plane is exposed for the HER evaluation, eliminating the contributions of the edge regions. Meanwhile, the micro-reactor configuration enables an accurate estimation of the electrochemical surface areas (ECSA) based on the exposed window region and the catalytic activities of simplex 2D surfaces. In order to eliminate the possible dissolution of the Pt electrode affecting the performance^29^, a graphite carbon rod was used as the counter electrode (Supplementary Fig. 17) in our set up. Moreover, no H_2_ bubble generation was found in the exposed windows to block the surface during testing^30^.

By comparing the polarization curves of monolayer pristine MoS_2_, FD-MoS_2_-3, FD-MoS_2_-5, and Pt-MoS_2_, the HER performances of their base planes are shown in Fig. 3c. As revealed, the pristine MoS_2_ base plane exhibits a quite poor HER activity because of its perfect crystal structure consisting of non-defective six-membered rings that lead to very few active sites, consistent with the previous experimental results^31^. In contrast, FD-MoS_2_-3, FD-MoS_2_-5, and Pt-MoS_2_ show enhanced HER activities than that of pristine MoS_2_, and particularly, FD-MoS_2_-5 is even superior, highlighting the advantages of Frenkel-defect engineering strategy on 2D materials.

The overpotential, one of the important indexes to evaluate electrochemical catalytic performances, is used to reflect the difficulty of HER (i.e. the external driving potential required under a rated current density). For example, at the current density of 10 mA cm^−2^, the base planes of FD-MoS_2_-3 (174 mV) and FD-MoS_2_-5 (164 mV) exhibit lower overpotentials than those of pristine MoS_2_ (358 mV) and Pt-MoS_2_ (211 mV). In addition, we further evaluated the HER performance of these materials based on multiple-measurement polarization curves (Supplementary Figs. 18–21). The results confirm that FD-MoS_2_-5 exhibits the best HER performance. Figure 3d shows the Tafel plots of these MoS_2_ electrocatalysts that are derived from the corresponding polarization curves^32^. Notably, FD-MoS_2_-5 (36 mV dec^−1^) and FD-MoS_2_-3 (89 mV dec^−1^) present much smaller Tafel slopes than those of pristine MoS_2_ (165 mV dec^−1^) and Pt-MoS_2_ (105 mV dec^−1^). Supplementary Figure 22 illustrates the plotting Tafel slopes corresponding to the onset potentials based on statistical analysis, showing that FD-MoS_2_-5 has the smallest value compared to Pt-MoS_2_. Considering their similar defect concentrations, this evidence highlights the efficient HER improvement from the Frenkel defects than Pt atom dopants in MoS_2_. Supplementary Figure 23 also shows the polarization curves of FD-MoS_2_-15 with more defected structures, whose HER performance was inferior to that of FD-MoS_2_-5. This indicates that increasing Frenkel defects may not continuously enhance the HER performance, which is also consistent with the results reported in the literature^8,33,34^. Besides, in previously reported works, the overpotential of monolayer MoS_2_ containing only S vacancies (or only Mo vacancies or both the two types of vacancies) at 10 mA cm^−2^ for HER was found to be ~230 mV (or in a range of 200–300 mV)^35–39^, all higher than that of our Frenkel-defected MoS_2_ catalysts, reflecting the advantages of the Frenkel defects over the Schottky defects in improving HER activities of MoS_2_ materials. Furthermore, chronoamperometry tests on the FD-MoS_2_-3 catalyst showed that the monolayer FD-MoS_2_-3 catalyst may sustain HER for ~10 min (Supplementary Fig. 24), representing a similar stability compared to the pristine monolayer MoS_2_.

### DFT calculations

**Fig. 4:**
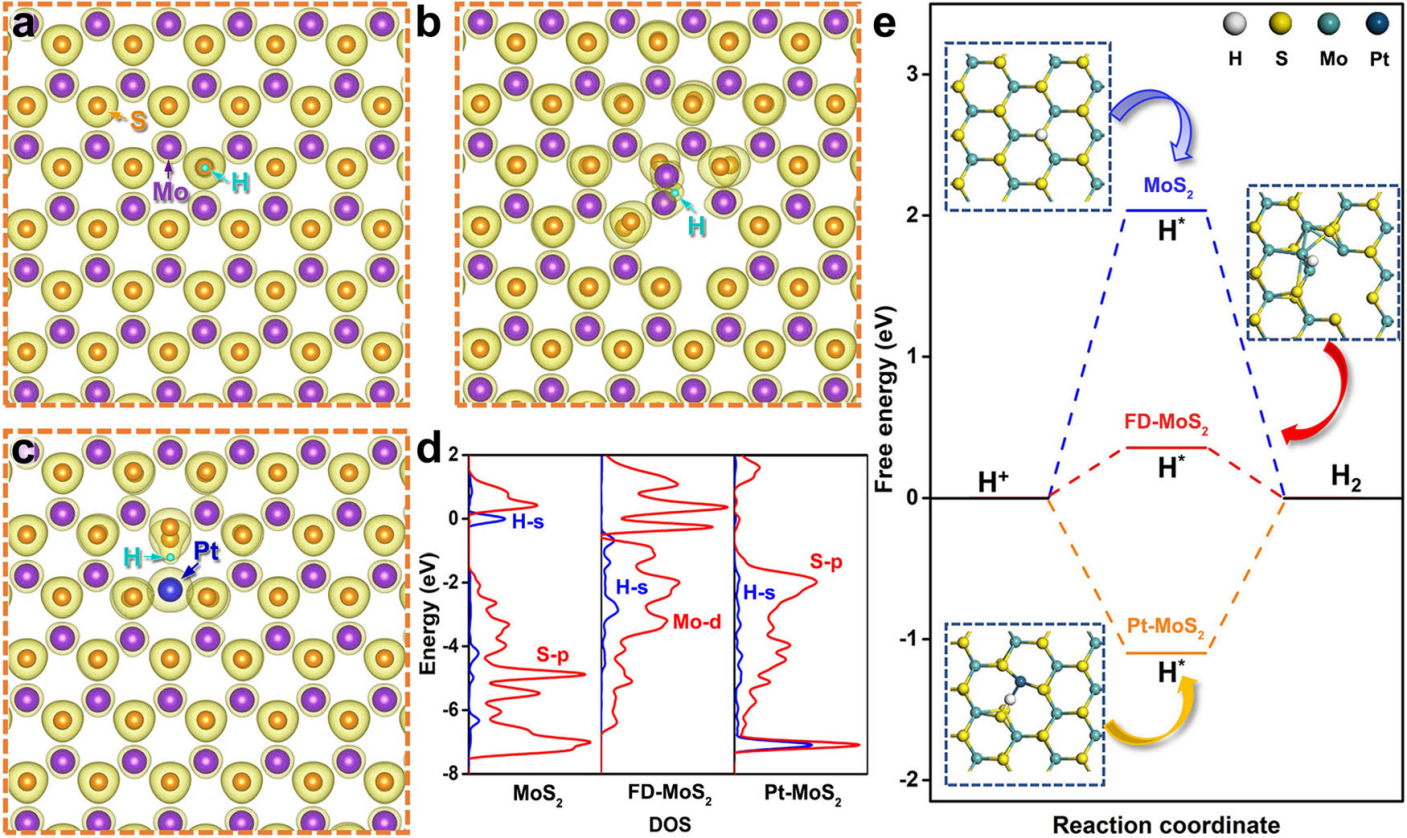
DFT calculation results of the surface charge density distributions and HER catalytic activities of monolayer pristine MoS_2_, FD-MoS_2_, and Pt-MoS_2_. **a**–**c** Calculated surface charge distributions when H is adsorbed on monolayer pristine MoS_2_, FD-MoS_2_, and Pt-MoS_2_, respectively. **d** Calculated PDOS of active site and H on the different catalyst surfaces. **e** Free energy diagrams of HER on the three MoS_2_ catalysts.

To gain deeper mechanistic insights into the efficient hydrogen evolution of the point-defected MoS_2_ catalysts, DFT calculation was performed to reveal the atomic outer valence electron charge density distributions of the models corresponding to monolayer pristine MoS_2_, FD-MoS_2_, and Pt-MoS_2_. As for the pristine MoS_2_, H is found to be adsorbed on top of the S site in the MoS_2_ surface plane, as illustrated in Fig. 4a. In contrast, on the surface plane of FD-MoS_2_, the migration of the Mo atom accompanying with the S vacancy modifies the surface charge distribution and offers additional charges between the S atoms and the Mo atoms adjacent to the original position. Hence, H prefers to adsorb in the vicinity of the interstitial Mo atom, more specifically, between the interstitial Mo atom and the nearby Mo atom (Fig. 4b). The high electron density around S in pristine MoS_2_ confirms the overly weaker binding for H* again, while the lower electron density around Mo in FD-MoS_2_ reveals a stronger binding ability. Besides, Fig. 4c presents the charge distribution of Pt-MoS_2_. The Pt single atoms that substitute the original Mo atoms provide larger charge densities at their exact lattice positions, and H is adsorbed between the Pt atom and the nearby S atom (Fig. 4c). It is illustrated that the charge density distributions of MoS_2_ change significantly with the introduction of different kinds of point defects, resulting in dissimilar hydrogen adsorption sites.

Furthermore, we compare the HER activities of pristine MoS_2_, FD-MoS_2_, and Pt-MoS_2_ base planes by projected density of states (PDOS) and energy calculations. Figure 4d, e show that the binding of H with the S atom on pristine monolayer MoS_2_ is quite weak, and the corresponding Gibbs free energy is 2.03 eV, indicating a difficult Volmer process and a poor activity for HER on well-defined MoS_2_ base plane. In contrast, a much stronger binding of H-S (−1.09 eV) is obtained on Pt-MoS_2_, where the electronic resonance from PDOS can be found at a lower energy level in Fig. 4d. Note that, the S-Pt bond is found to be broken with the adsorbed H on Pt-MoS_2_. Importantly, the adsorption energy of H* on Mo in FD-MoS_2_ is calculated to be 0.36 eV, implying a moderate and suitable binding energy for HER on the studied surfaces. In addition, we further analyzed the different active sites in FD-MoS_2_, and the results showed that interstitial Mo atoms are the optimal HER active centers (Supplementary Fig. 25). Therefore, based on the DFT calculation, the HER activity of the MoS_2_ models should follow the sequence, FD-MoS_2_ > Pt-MoS_2_ > MoS_2_, consistent with our experimental evidence. Meanwhile, we also calculated the H* adsorption energy of FD-MoS_2_ with different defect concentrations (Supplementary Fig. 26), and the results indicated that a little bit higher concentrations may be beneficial to the HER catalytic activity, in agreement with our experimental results of FD-MoS_2_-3 and FD-MoS_2_-5 (Fig. 3c, d). Besides, we further analyzed the partial electron density distribution of the Frankel defect structure in MoS_2_ (Supplementary Fig. 27). The formation of Frankel defects causes more electrons to gather in its vicinity, making the defects become active sites for catalyzing the HER.

## Discussion

In summary, a Frenkel-defected monolayer MoS_2_ catalyst has been designed and synthesized via a facile Ar annealing treatment, and the atomic configurations of the Frenkel defects in MoS_2_ are revealed by AC-STEM observation. According to the electrochemical test employing the micro-reactor, FD-MoS_2_ shows an enhanced HER activity compared to the pristine MoS_2_ and Pt-MoS_2_ catalysts. DFT calculation reveals the unique charge distribution introduced by the existence of Frenkel defects in MoS_2_, which change the H adsorption site and provide a moderate adsorption energy of H* for FD-MoS_2_. This work not only gains insight into the structure-property relationships of point-defected MoS_2_ materials, but also provides an efficient and economical defect engineering strategy over 2D electrochemical catalysts at atomic level.

## Methods

### Chemicals

The SiO_2_/Si (270 nm SiO_2_) substrates were purchased from Suzhou Ruicai Semiconductor Co., Ltd.; MoO_3_ (≥99.5%) and S powder (≥99.5%) were purchased from Sigma-Aldrich (Shanghai) Trading Co., Ltd.; NaCl (≥99.5%), chloroplatinic acid and sodium molybdate (99.0%) were purchased from Shanghai Aladdin Biochemical Technology Co., Ltd.

### Synthesis of monolayer pristine MoS_2_

Firstly, the SiO_2_/Si substrates were cleaned by following the sequence of using piranha solution, isopropanol, and DI water^40^. Then MoO_3_ (15 mg) and NaCl (3 mg) were mixed and placed in the middle of an aluminum trioxide crucible. The SiO_2_/Si substrate (size: 1 × 2 cm) was located above the powders, and the crucible was placed in the middle of a quartz tube in the CVD furnace. Sulfur powder (320 mg) was placed in another crucible in upstream, ~15 cm away from the middle of the furnace. The system was firstly ventilated with Ar (300 sccm) for 10 min for flushing, and then Ar (100 sccm) was maintained to flow in the system. The furnace was heated up to 705 °C in 40 min, and then maintained at 705 °C for 5 min. At the end, the CVD furnace was cooled to room temperature naturally.

### Synthesis of monolayer FD-MoS_2_

The grown pristine MoS_2_ was placed in the middle of an additional quartz tube. The system was first ventilated with Ar (300 sccm) for 10 min to remove the impurities in the tube, and the inert atmosphere was maintained with Ar (100 sccm) to flow in the system. Then, the furnace was heated to the specified temperature (400 °C) within 60 min in this atmosphere, and kept at 400 °C for 3 min. Finally, the CVD furnace was slowly cooled to room temperature. The synthesis conditions of FD-MoS_2_-5 and FD-MoS_2_-15 are similar to that of FD-MoS_2_-3, except the extended annealing time of 5 and 15 min, respectively.

### Synthesis of monolayer Pt-MoS_2_

Pt-doped MoS_2_ was obtained according to our previously reported method by the following steps^41^. First, 995 μL of Na_2_MoO_4_ (10 mmol) and 5 μL of H_2_PtCl_6_ (24.4 mmol) were mixed to form a precursor solution. Fresh SiO_2_/Si substrates were treated under oxygen plasma (60 W) for 1 min (CIF Tech Co., Ltd. CPC-C-40 KHz). The precursor solution was spin-coated onto the treated substrates at 3000 rpm for 1 min. Then, the substrate with precursor was loaded into the center of the furnace. A crucible with sulfur powder (150 mg) was located at upstream, 15 cm away from the middle of the furnace. Ar (270 sccm) was used as carrying gas, and the growth temperature was gradually raised to 850 °C in 40 min. The sulfur powder was subsequently pushed into the hot zone and kept for 5 min at 850 °C. Then the CVD furnace was slowly cooled to room temperature.

### Structural characterization

The morphologies of these 2D samples were observed by optical microscopy (Nikon H600L) and atomic force microscope (AFM, Bruker Dimension Icon). Raman and PL spectra were obtained by Horiba Instruments INC (1024×256-OE) equipped with a 532 nm laser excitation and a CCD detector in a backscattering geometry. The XPS data of these 2D samples on the SiO_2_/Si substrates were analyzed by ESCALAB 250Xi X-ray photoelectron spectroscopy equipped with a monochromatic Al Kα source (*λ* = 1486.6 eV). The ∼284.8 eV adventitious C 1 s peak was used for charging corrections. The AC-STEM characterization was performed using a ThermoFisher Themis Z microscope equipped with two aberration correctors under 300 kV.

### Device fabrication

According to our previous device fabrication method^41^, the SiO_2_/Si substrates with different 2D products were spin-coated with SPR-220-3a photoresist at 4000 rpm for 1 min, and then baked at 115 °C for 90 s. Next, the SiO_2_/Si substrates covered with photoresist were patterned for electrode pattern by ultraviolet, and then deposited with In/Au (10 nm/50 nm) by thermal evaporation to connect the monolayer 2D samples. Residual photoresist was removed by acetone following a lift-off process, and the metallic electrode patterns were obtained for devices. In the microelectrocatalytic process, photoresist was spin-coated again on the evaporated devices in SiO_2_/Si substrates, and baked at 115 °C for 90 s. And then these 2D samples exposed with proper ultraviolet beam to open windows (the actual surface area of the exposed 2D materials) on the target positions of the monolayer samples for HER measurements.

### HER measurements

The micro-electrocatalysis performance was tested by a three-electrode system using a CHI 660E electrochemical workstation. Graphite carbon electrode with a diameter of 1 mm tip served as the count electrode, and a homemade saturated Ag/AgCl electrode served as the reference electrode. The exposed area of each measured monolayer 2D samples served as the working electrode. The HER activity of each monolayer was evaluated in 0.5 M H_2_SO_4_ electrolyte by linear sweep voltammetry at a scan rate of 5 mV s^−1^. The volume of the droplets on the window surface of the 2D material was fixed at 5 μL. All reported potentials were converted to reversible hydrogen electrode (RHE) potentials, and no iR-corrected was used in all the electrochemical measurements.

### Computational methods

DFT calculation was performed using the Vienna ab initio simulation package (VASP)^42^. The Generalized Gradient Approximation (GGA)^43^ with the Perdew–Burke–Ernzerhof (PBE) functional was used for all the calculations. HER performance and fundamental electronic characters were studied over the three different catalysts, including monolayer pristine MoS_2_, Pt-MoS_2_ and FD-MoS_2_. Each of the structures was built based on a periodic 6 × 6 single layer MoS_2_ surface with 36 Mo atoms and 72 S atoms (up to 144 Mo atoms and 288 S atoms were built). A Monkhorst-Pack k-point 2 × 2 × 1 was used for all the surface calculations with the cut-off energy of 400 eV. The structural optimization was conducted using the conjugate-gradient (CG) algorithm^44^, and the force convergence was set to be 0.05 eV ∙ Å^−1^. The standard free energies correction^45^ was used to obtain the free energy at 298 K, following $\triangle G=\triangle E+\triangle {ZPE}+\triangle U-T\triangle S$, where *G, E, ZPE, U, T*, and *S* refer to the free energy, total energy, zero-point energy, inner energy, temperature and entropy, respectively. In addition, electrical neutrality was considered in all DFT calculations. Notably, due to the large size of the MoS_2_ model, only GGA could be utilized here. Generally, more accurate electronic structure information could be calculated by using a higher-precision or hybrid functional.

## Supplementary information

Supplementary Information

## Data Availability

All data supporting the findings of this study are available from the Source Data. Source data are provided with this paper.

## Source data

Source Data
