# Supplementary material for: Frenkel-defected monolayer MoS2 catalysts for efficient hydrogen evolution
Source: Nat Commun. 2022 Apr 22;13:2193. doi: 10.1038/s41467-022-29929-7 (PMC9033855; doi:10.1038/s41467-022-29929-7)
Supplement: Supplementary file 1 — Supplementary Information [file 41467_2022_29929_MOESM1_ESM.pdf]

# Supplementary Information for

Frenkel-defected monolayer $MoS_2$ catalysts for efficient hydrogen evolution

Jie Xu[1,#], Gonglei Shao[2,3,#], Xuan Tang[4,#], Fang Lv[1], Haiyan Xiang[2], Changfei Jing[5], Song Liu[2*], Sheng Dai[4*], Yanguang Li[1*], Jun Luo[5*], Zhen Zhou[3]

[1] Institute of Functional Nano & Soft Materials (FUNSOM), Jiangsu Key Laboratory for Carbon-Based Functional Materials and Devices, Soochow University, Suzhou 215123, China.

[2] State Key Laboratory of Chemo/Biosensing and Chemometrics, College of Chemistry and Chemical Engineering, Hunan University, Changsha 410082, Hunan, China.

[3] Engineering Research Center of Advanced Functional Material Manufacturing of Ministry of Education, School of Chemical Engineering, Zhengzhou University, Zhengzhou 450001, China.

[4] Feringa Nobel Prize Scientist Joint Research Centre, School of Chemistry and Molecular Engineering, East China University of Science & Technology, Shanghai 200237, China.

[5] School of Materials Science and Engineering, Tianjin Key Lab of Photoelectric Materials & Devices, Tianjin University of Technology, Tianjin 300384, China.

[#] The three authors contributed equally: Jie Xu, Gonglei Shao, Xuan Tang.

*Correspondence to: liusong@hnu.edu.cn; shengdai@ecust.edu.cn; yanguang@suda.edu.cn; jluo@tjut.edu.cn

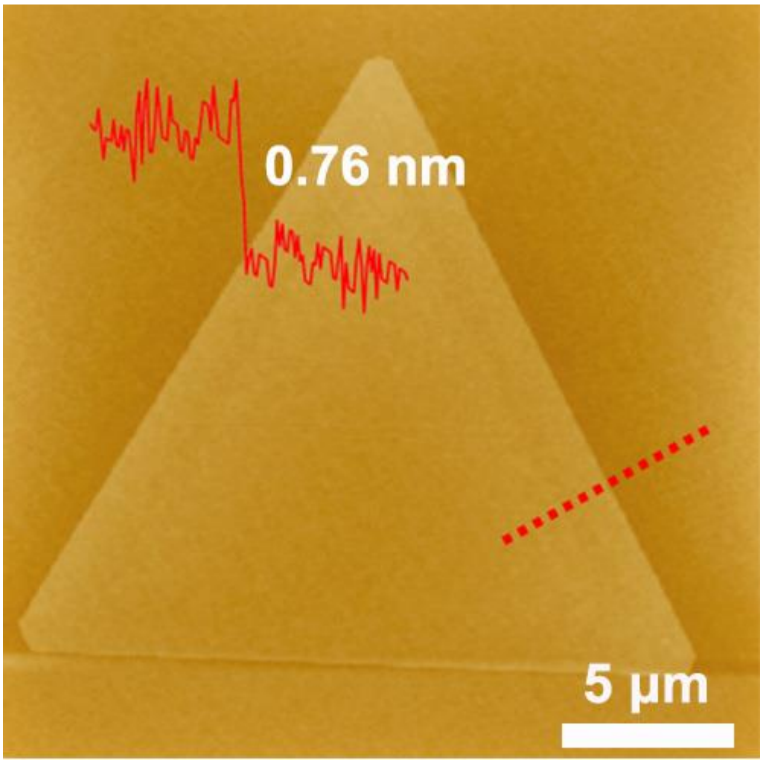


**Supplementary Fig. 1** AFM characterization of monolayer FD-$MoS_2$-3 catalyst. AFM image and the corresponding height profile along the dashed line with momolayer thickness of 0.76 nm.

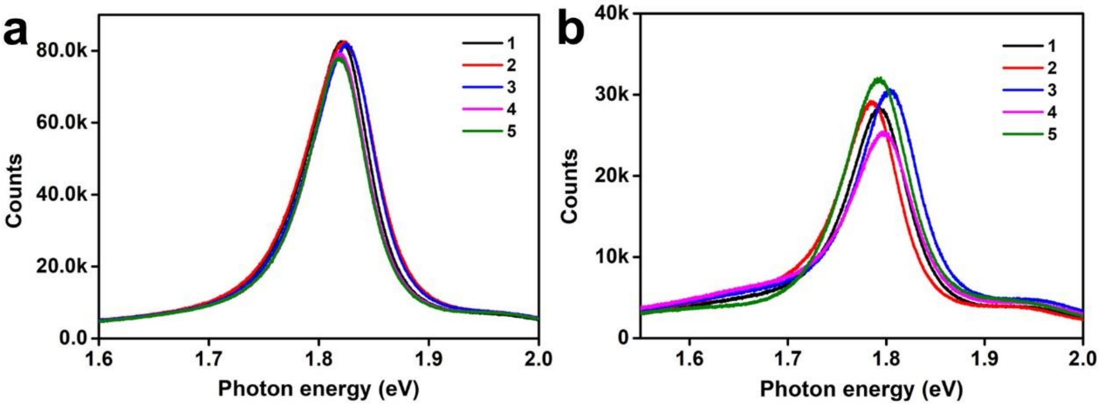


**Supplementary Fig. 2** PL characteriaiton of pristine $MoS_2$ and FD-$MoS_2$-3 catalysts. PL spectra taken from five different regions of pristine $MoS_2$ (a) and FD-$MoS_2$-3 (b) catalysts. The average value of the peak counts and the corresponding standard deviation in prisitne $MoS_2$ is 81376±1738. The average value of the peak counts and the corresponding standard deviation in the PL spectrum of FD-$MoS_2$-3 is 28937±2493. This indicates that the peak intensity in the PL spectrum of FD-$MoS_2$-3 is significantly weaker than that of prisitne $MoS_2$, and the band gap of FD-$MoS_2$-3 (1.80 eV) shows red-shifts compare to that of prisitne $MoS_2$ (1.82 eV).

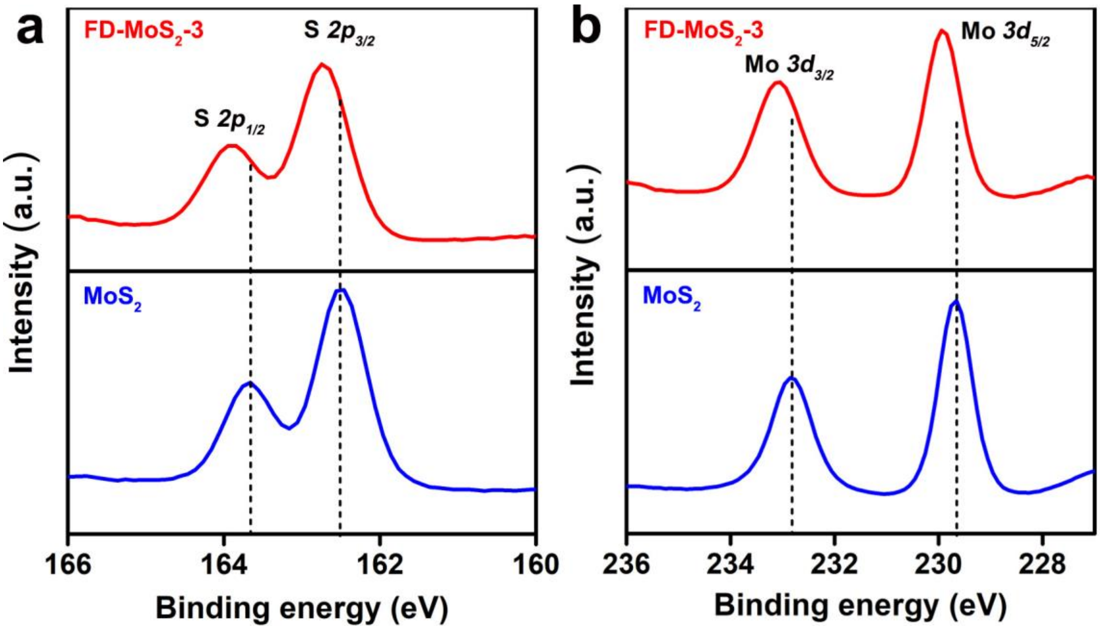


**Supplementary Fig. 3** XPS spectra of monolayer pristine $MoS_2$ and FD-$MoS_2$-3. (a) Deconvoluted S *2p* spectra of pristine $MoS_2$ and FD-$MoS_2$-3. (b) Deconvoluted Mo *3d* spectra of pristine $MoS_2$ and FD-$MoS_2$-3.

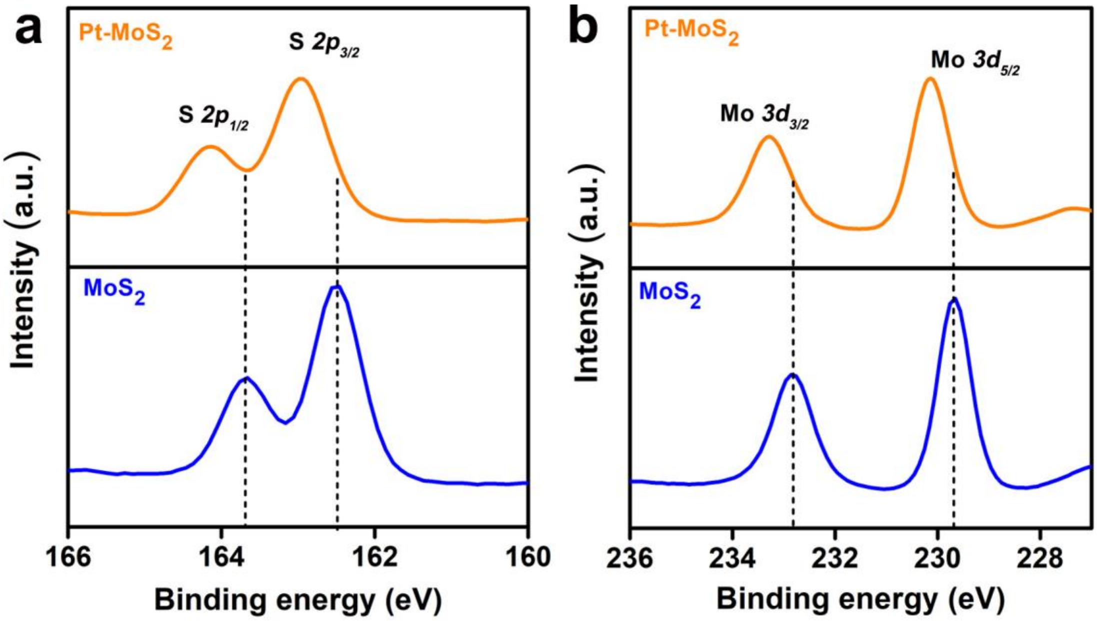


**Supplementary Fig. 4** XPS spectra of monolayer pristine $MoS_2$ and Pt-$MoS_2$. (a) Deconvoluted S *2p* spectra of pristine $MoS_2$ and Pt-$MoS_2$. (b) Deconvoluted Mo *3d* spectra of pristine $MoS_2$ and Pt-$MoS_2$.

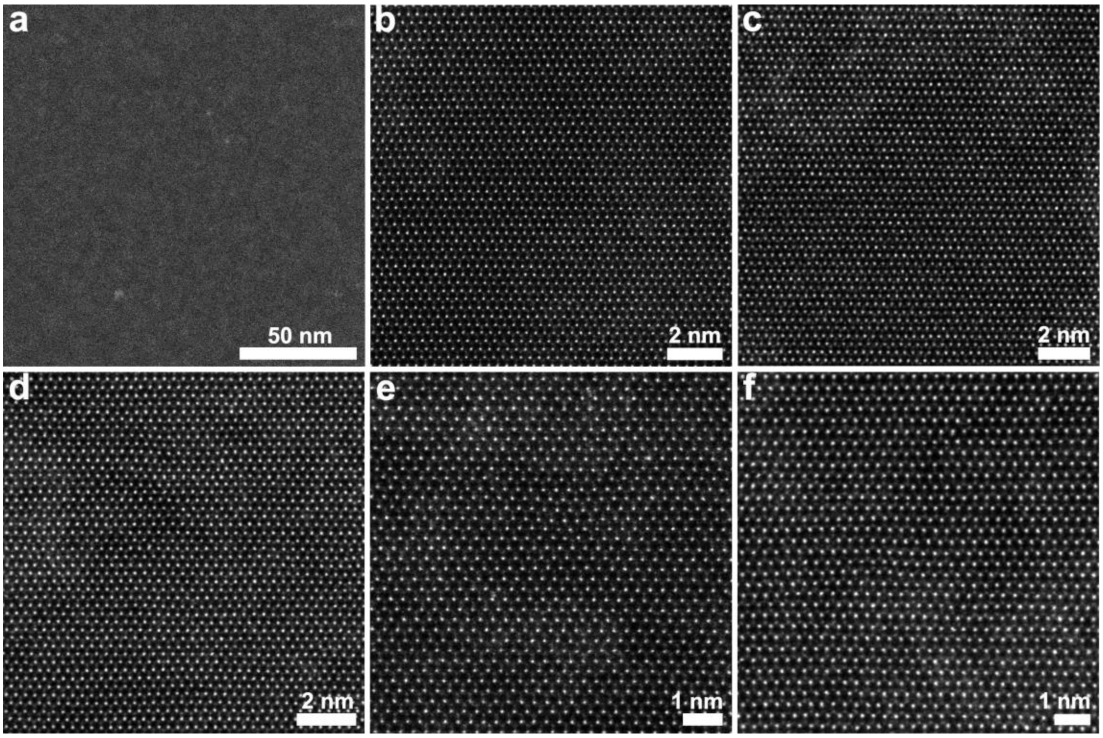


**Supplementary Fig. 5** AC-STEM characterization of monolayer pristine $MoS_2$ catalysts. (a) The low-magnification HAADF-STEM image of monolayer pristine $MoS_2$. (b-f) The atomic-resolution HAADF-STEM images in different regions of pristine $MoS_2$. These areas are randomly picked up from the monolayer pristine $MoS_2$, and no obvious point defects are found from HAADF-STEM observation.

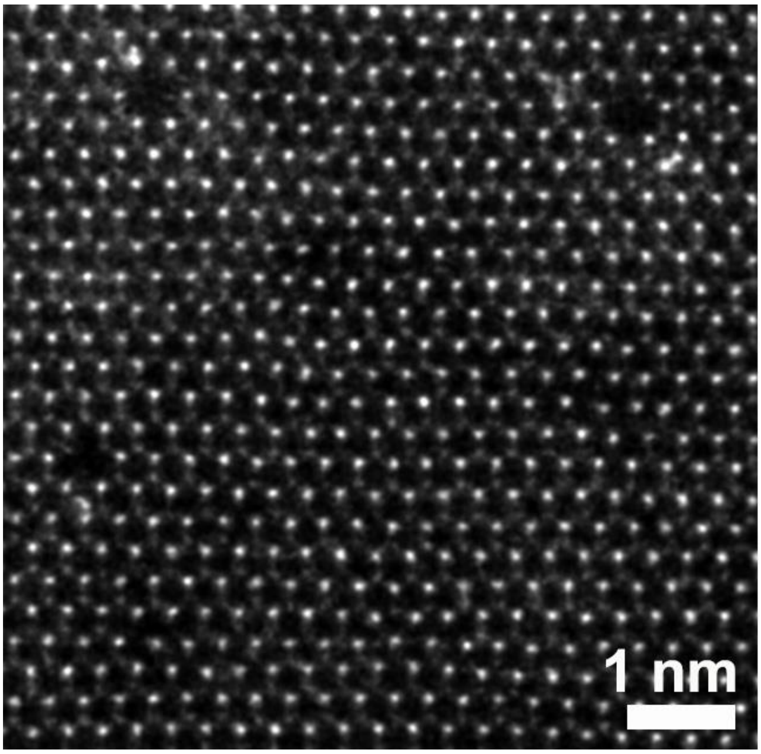


**Supplementary Fig. 6** Atomic-resolution HAADF-STEM image of FD-$MoS_2$-3. The existence of Frankel defects can be found. This image is the raw data of Figure 2d.

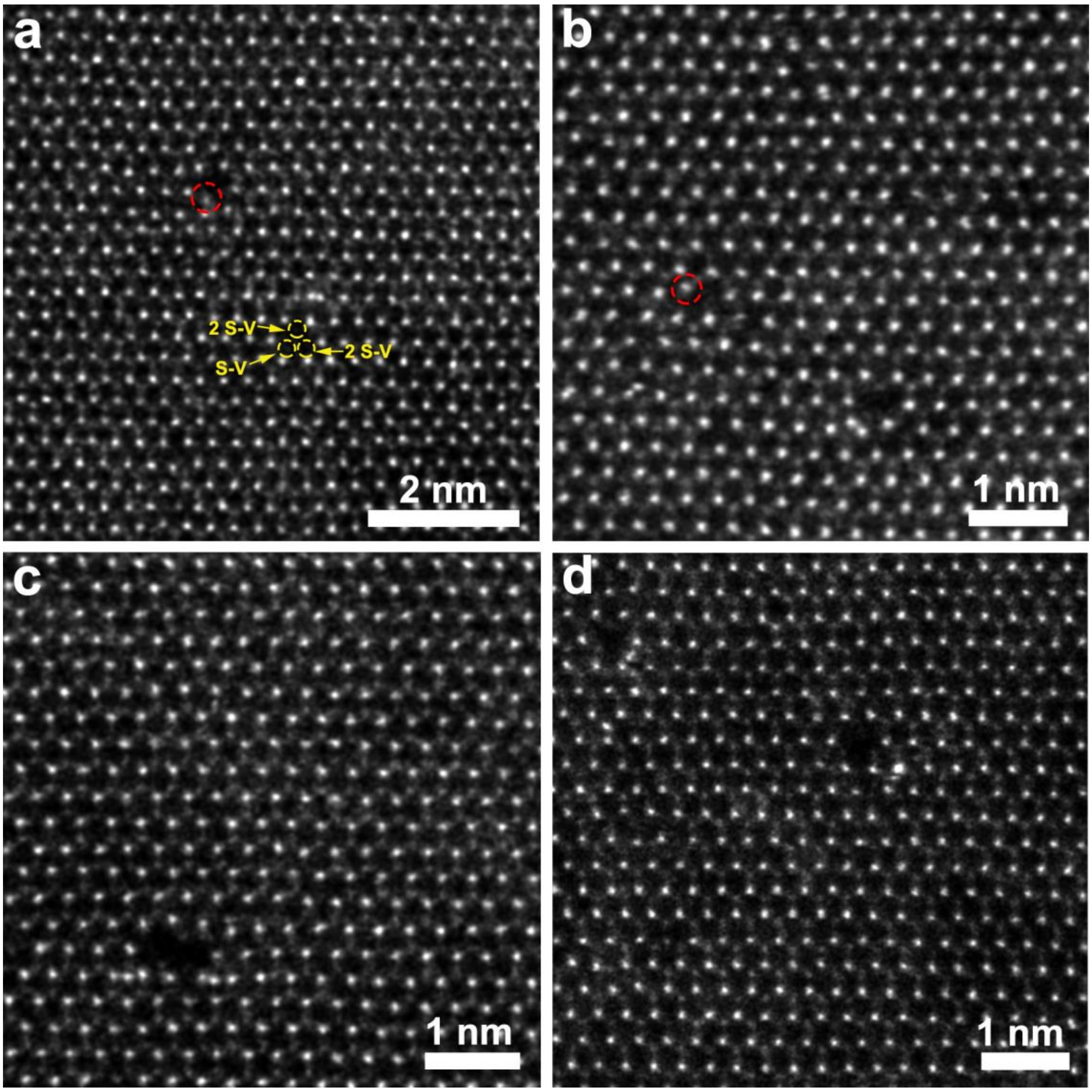


**Supplementary Fig. 7** AC-STEM characterization of FD-$MoS_2$-3 catalysts. (a-d) Typical atomic-resolution HAADF images of different regions of FD-$MoS_2$-3. These areas in (a-d) were selected randomly, and the Frankel defects can be found in these images. More importantly, supplementary Figs. 7a and 7b show the intermediate state of interstitial Mo atoms (red dashed circles) leaving their original positions and migrating to nearby Mo atoms. In addition, the concentrations of Frankel defects corresponding to a-d and Figure 2d in the manuscript are 0.36%, 0.56%, 0.53%, 0.50% and 0.57%, respectively. The overall concentration of Frankel defects in FD-$MoS_2$-3 is approximately 0.50% in average.

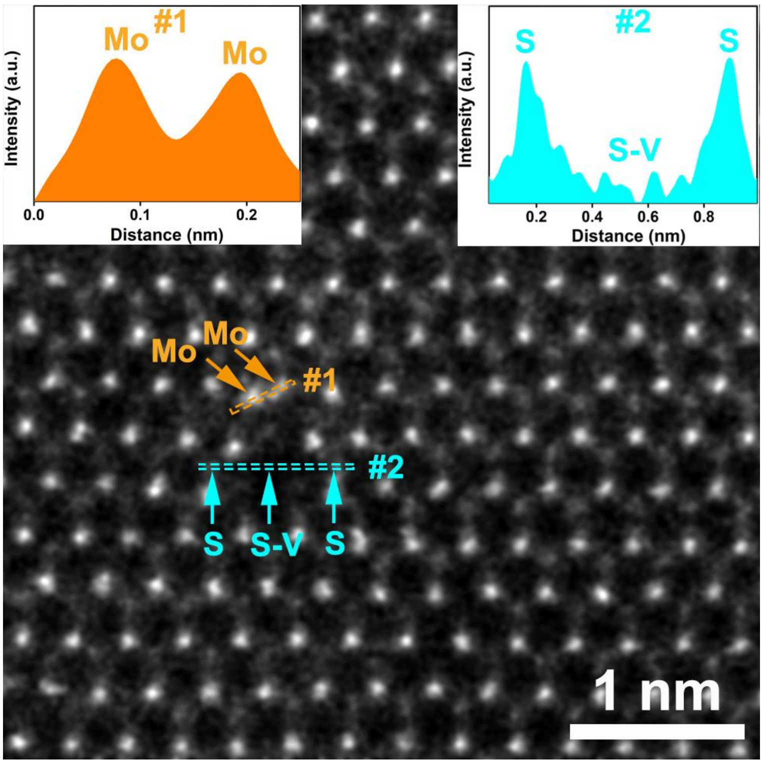


**Supplementary Fig. 8** Atomic-resolution HAADF-STEM image of monolayer FD-$MoS_2$-3. Intensity analysis showing the interstitial Mo and regular Mo atoms (inset #1), S atoms (S), and S vacancies (S-V) (inset #2).

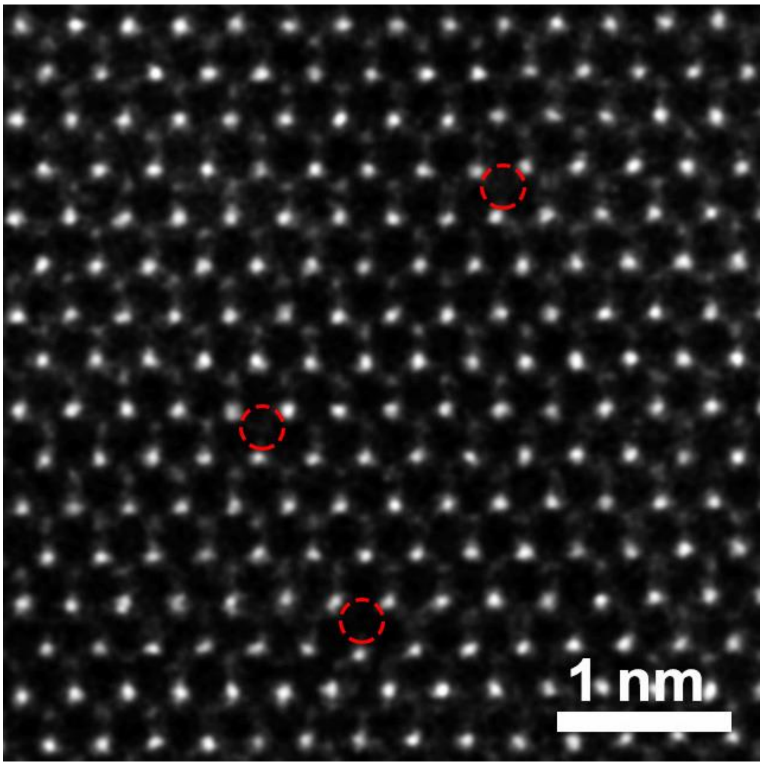


**Supplementary Fig. 9** Atomic-resolution HAADF-STEM image of monolayer pristine $MoS_2$ that was annealed in Ar atmosphere at 400 °C for 1 min. The red dashed circles represent the S vacancies.

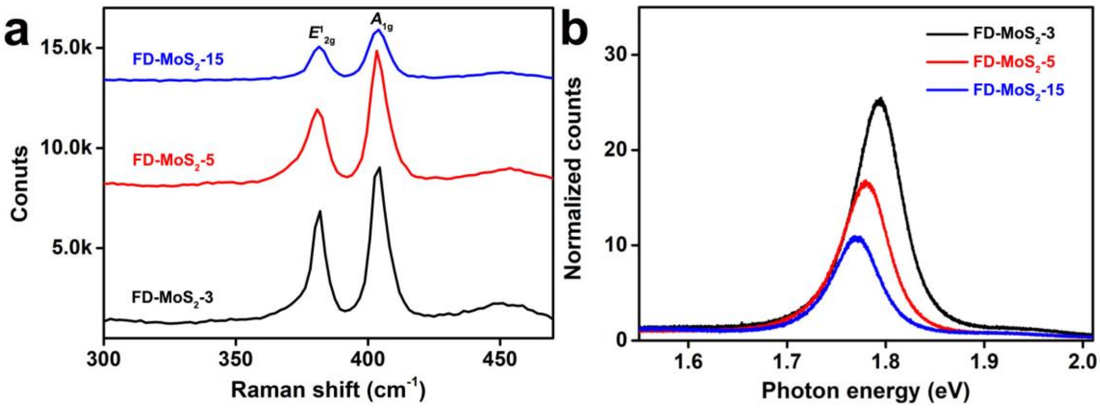


**Supplementary Fig. 10** Raman and PL characterization of FD-$MoS_2$-3, FD-$MoS_2$-5, and FD-$MoS_2$-15. (a) The Raman spectra of FD-$MoS_2$-3, FD-$MoS_2$-5, and FD-$MoS_2$-15. Compared with FD-$MoS_2$-3, the peaks of FD-$MoS_2$-5 and FD-$MoS_2$-15 show a wider FWHM, indicating more defective structures. (b) The normalized PL spectra of FD-$MoS_2$-3, FD-$MoS_2$-5, and FD-$MoS_2$-15. Compared with FD-$MoS_2$-3, the main peak intensities of FD-$MoS_2$-5 and FD-$MoS_2$-15 are weakened, also indicating that more defects are formed.

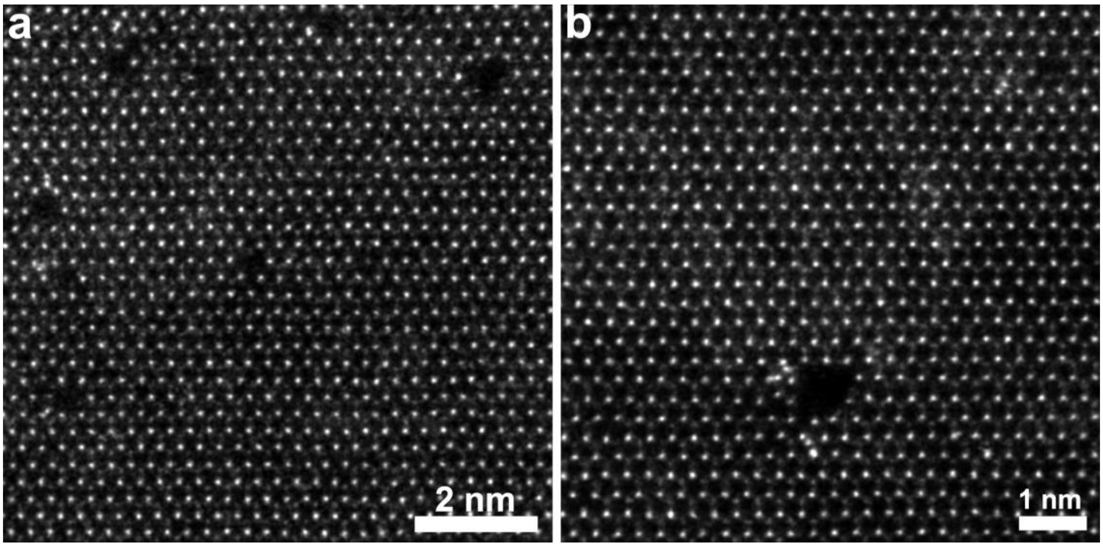


**Supplementary Fig. 11** AC-STEM characterization of FD-$MoS_2$-5 catalysts. (a,b) Typical atomic-resolution HAADF-STEM images of FD-$MoS_2$-5. Compared with FD-$MoS_2$-3, more Frankel defects are observed. The concentrations of Frankel defects in (a) and (b) are 0.95% and 0.75%, respectively. The overall concentration of Frankel defects in FD-$MoS_2$-5 is approximately 0.85% in average.

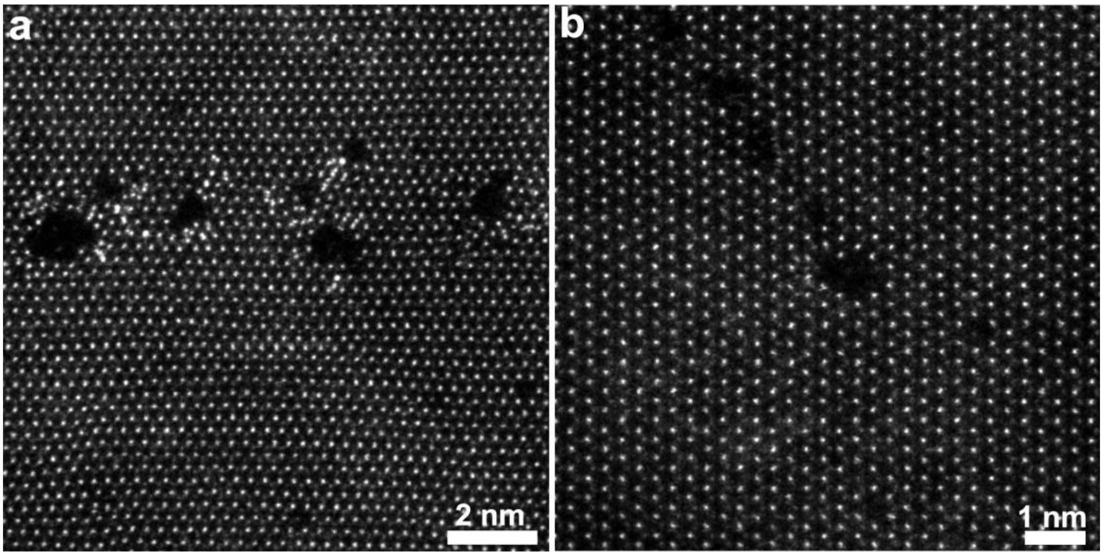


**Supplementary Fig. 12** Atomic-resolution HAADF-STEM images of FD-$MoS_2$-15. Compared with FD-$MoS_2$-3, many hole defects are observed in addition to the Frankel defects (a,b).

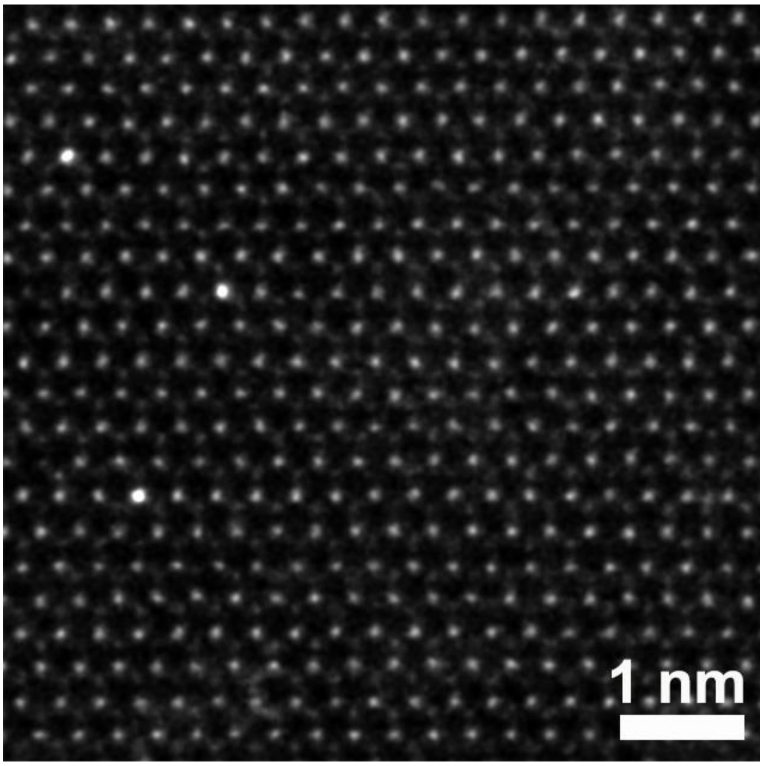


**Supplementary Fig. 13** Atomic-resolution HAADF-STEM image of Pt-$MoS_2$ catalyst. Pt single atoms showing highest Z-contrast can be identified. This is the raw data of Figure 2g.

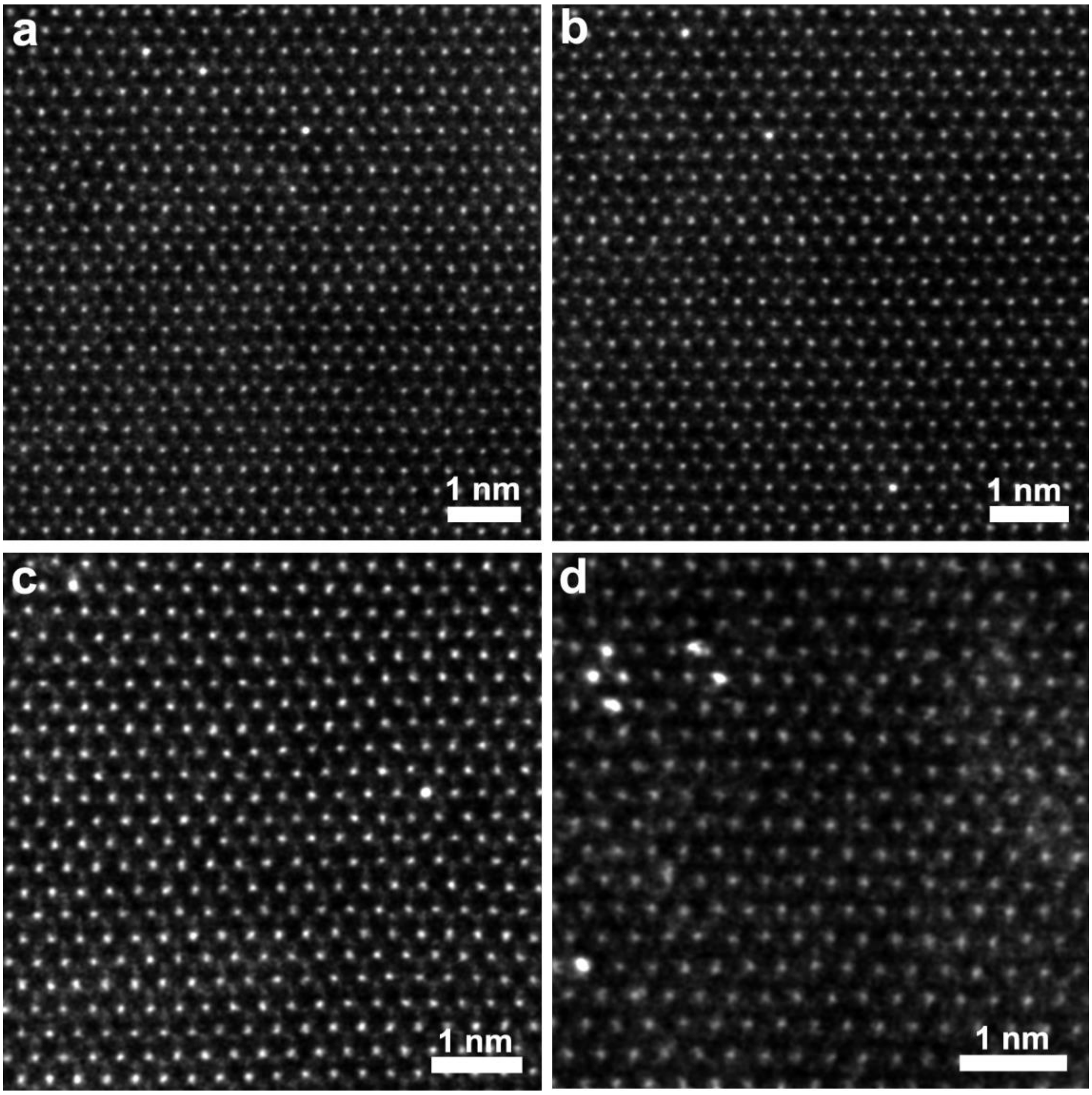


**Supplementary Fig. 14** AC-STEM characterization of Pt-$MoS_2$ catalysts. (a-d) Typical atomic-resolution HAADF-STEM images in different regions of Pt-$MoS_2$. These areas are randomly pick up from the monolayer Pt-$MoS_2$. The concentrations of Pt single-atom doping in (a-d) and Figure 2g are 0.48%, 0.54%, 0.43%, 1.86%, and 0.69%, respectively. The overall concentration of Pt doping in Pt-$MoS_2$ catalysts is approximately 0.80% in average.

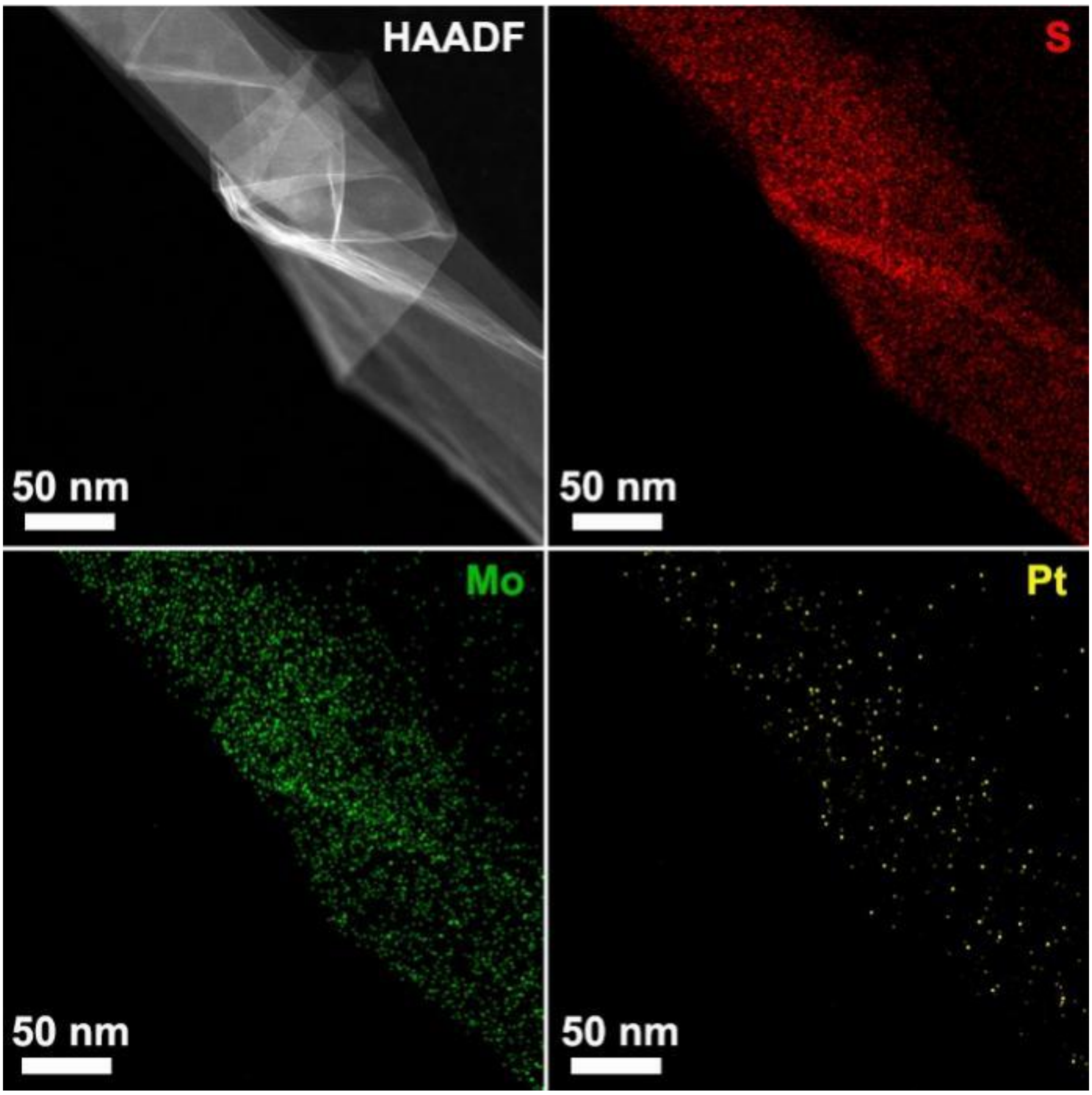


**Supplementary Fig. 15** HAADF-STEM and EDS characteration of Pt-$MoS_2$. Representative HAADF-STEM image and the corresponding EDS elemental maps of S, Mo, and Pt. The results show the uniform Pt distribution on $MoS_2$.

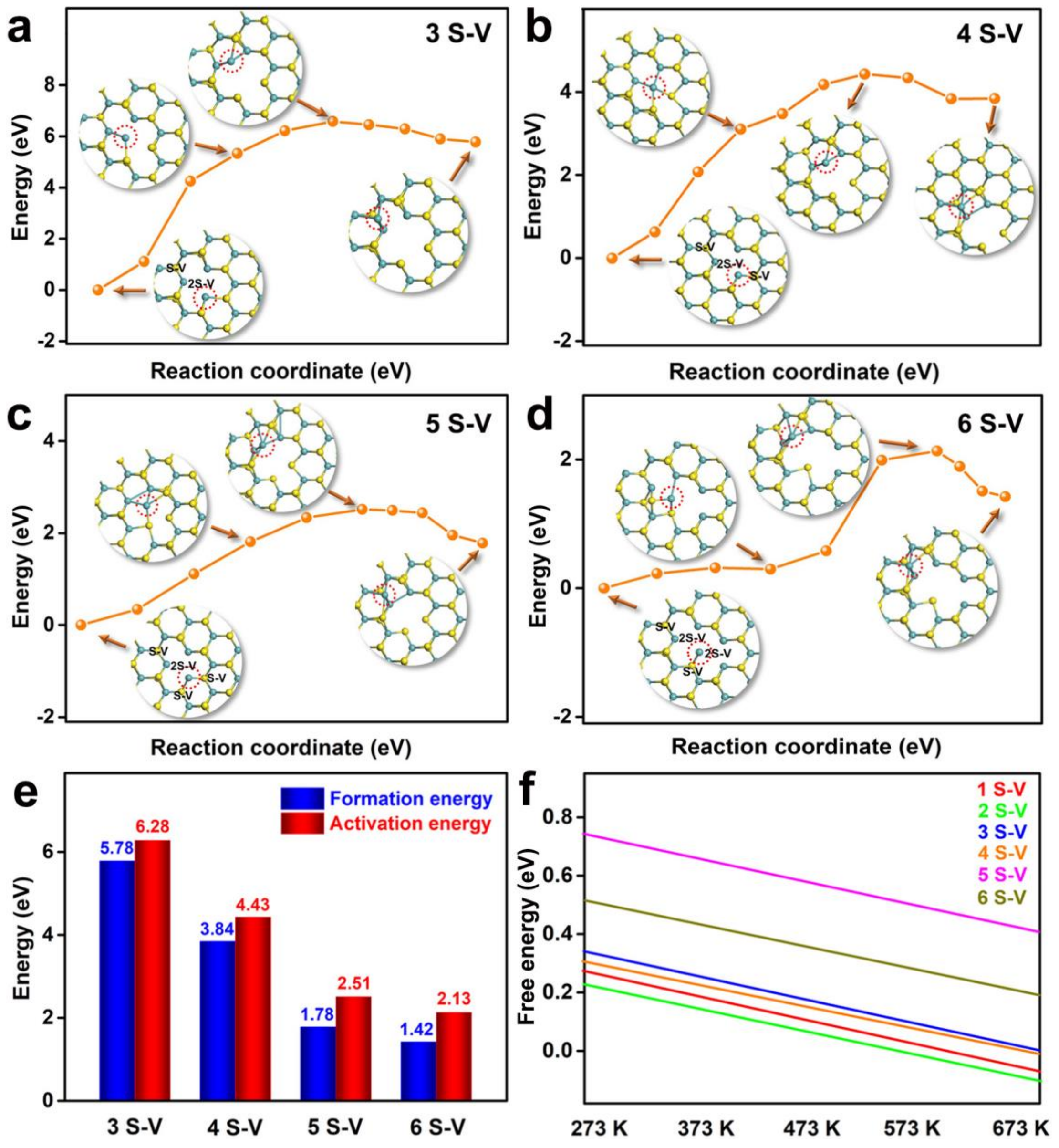


**Supplementary Fig. 16** Energy change of Frenkel defects formed in monolayer $MoS_2$ with different S vacancies (S-V) by DFT calculation. (a-d**)** Reaction path diagrams of Frenkel defect formation in FD-$MoS_2$ with different quantities of S-V. The optimal path to form Frenkel defects with different S vacancies was obtained by the DFT based CI-NEB calculation method on the basis of total energy. (e) Frenkel defect formation energy and activation energy with different S-V in the local area (see initial images in a-d). (f) Different S vacancy formation energy of pristine $MoS_2$ at different

temperatures. The calculation method for the free energy of different numbers of S vacancies is formation energy = $E_{surf(n)} + E_{S\text{-}reference} - E_{surf(n\text{-}1)}$, where surf denotes the $MoS_2$ surface, S-reference denotes the $SO_2$-$O_2$, and n denotes the number of S vacancies in the system. The results reveal that the formation energy decreases from 5.78 eV to 3.84 eV (from 6.58 eV to 4.43 eV for the barrier) as the S vacancies change from four to three in the localized region in $MoS_2$. Furthermore, we investigated the formation energy of Frenkel defects in the cases with more S vacancies. The results show that when there are five and six S vacancies in the local area, the formation energy of Frenkel defects is reduced to 1.78 eV and 1.42 eV, respectively. In addition, the formation of multiple S vacancies is also thermodynamically feasible at 673 K. These calculations showed that at relatively high formation energy, Frenkel defects can also be formed mainly due to the process of multi-S vacancies and high temperature treatment.

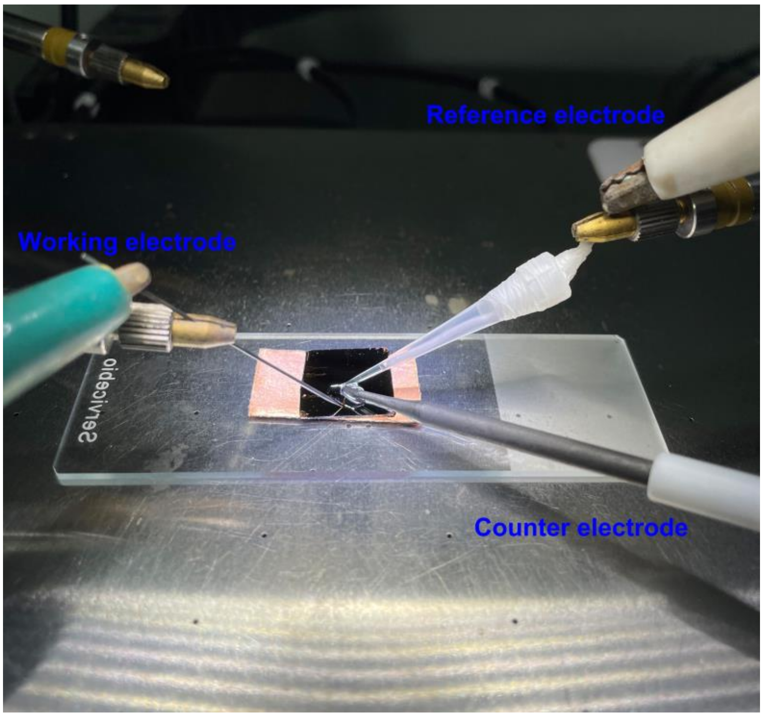


**Supplementary Fig. 17** Optical microscopy image of microelectrochemical devices. Upper right: A homemade saturated Ag/AgCl electrode serve as the reference electrode. Bottom right: Graphite carbon electrode with a diameter of 1 mm tip serve as the count electrode. Left: The exposed area of each measured monolayer 2D samples connected with probe and electrode serve as the working electrode.

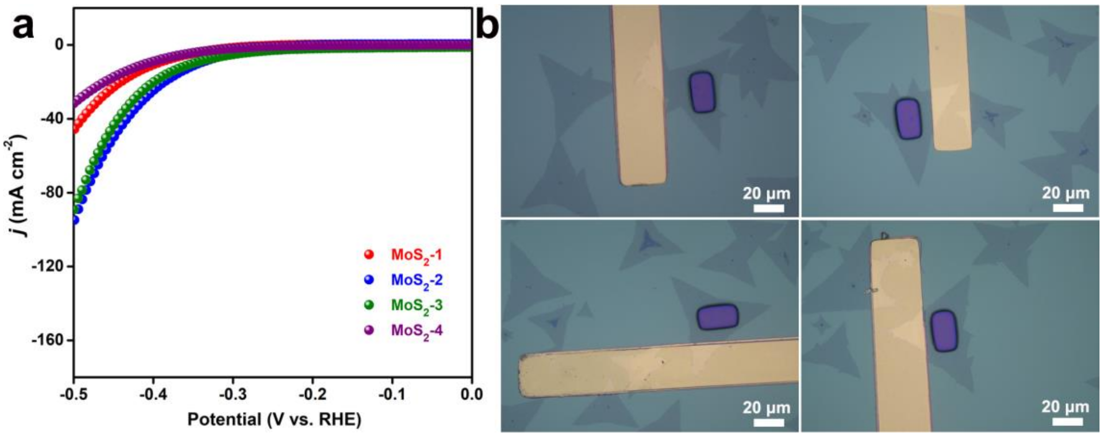


**Supplementary Fig. 18** HER performance of monolayer pristine $MoS_2$ catalysts and corresponding optical microscopy images. (a) Polarization curves of different sample areas of monolayer pristine $MoS_2$ in 0.5 M $H_2SO_4$. All the current values are normalized by the exposed $MoS_2$ surface areas. (b) The corresponding optical microscopy images of exposure areas of different pure $MoS_2$ samples. The purplish red part is the exposed pure $MoS_2$ base surface with an area of (15 μm×30 μm) 450 $μm^2$.

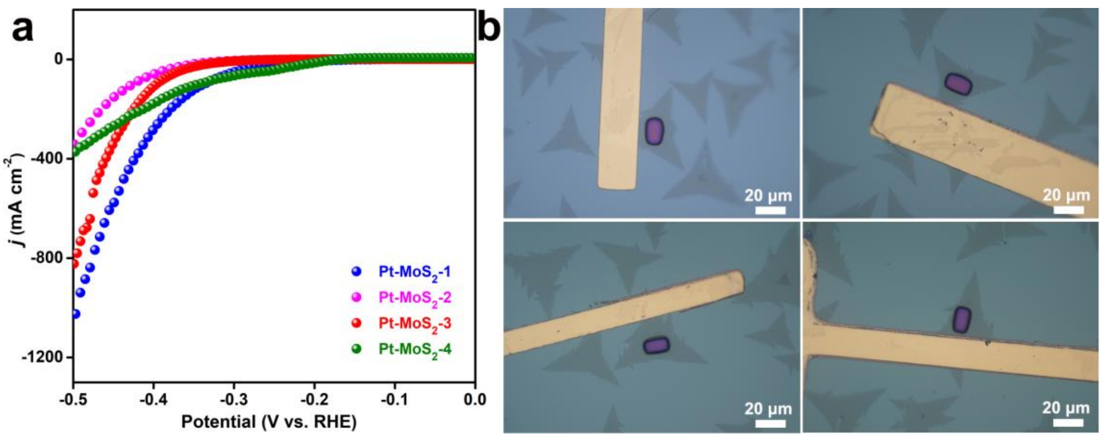


**Supplementary Fig. 19** HER performance of monolayer Pt-$MoS_2$ catalysts and corresponding optical microscopy images. (a) Polarization curves of different sample areas of monolayer Pt-$MoS_2$ in 0.5 M $H_2SO_4$. All current values are normalized by the exposed Pt-$MoS_2$ surface areas. (b) The corresponding optical microscopy images of exposure areas of different Pt-$MoS_2$ samples. The purplish red part is the exposed Pt-$MoS_2$ base surface with an area of (10 μm×20 μm) 200 μm$^2$.

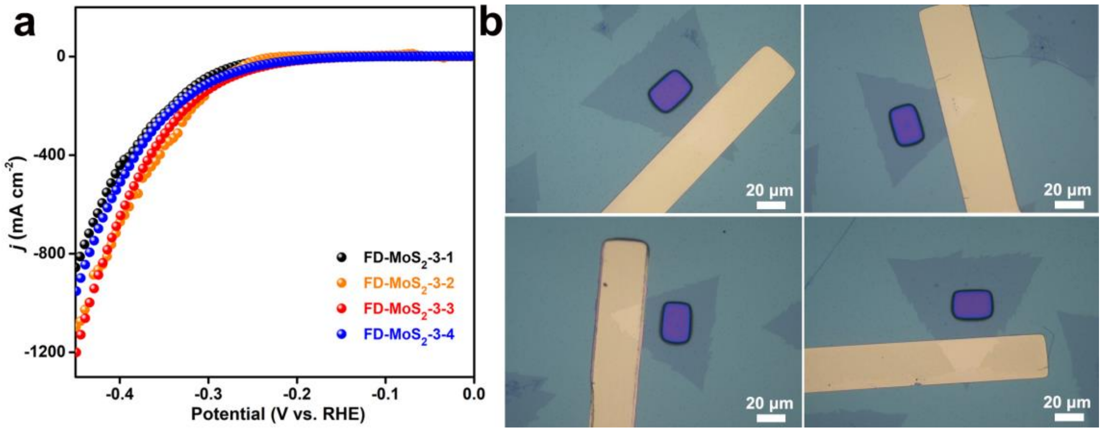


**Supplementary Fig. 20** HER performance of monolayer FD-$MoS_2$-3 catalysts and corresponding optical microscopy images. (a) Polarization curves of different sample areas of monolayer FD-$MoS_2$-3 in 0.5 M $H_2SO_4$. All current values are normalized by the exposed FD-$MoS_2$-3 surface areas. (b) The corresponding optical microscopy images of exposure areas of different FD-$MoS_2$-3 samples. The purplish red part is the exposed FD-$MoS_2$-3 base surface with an area of (20 μm×30 μm) 600 $\mu m^2$.

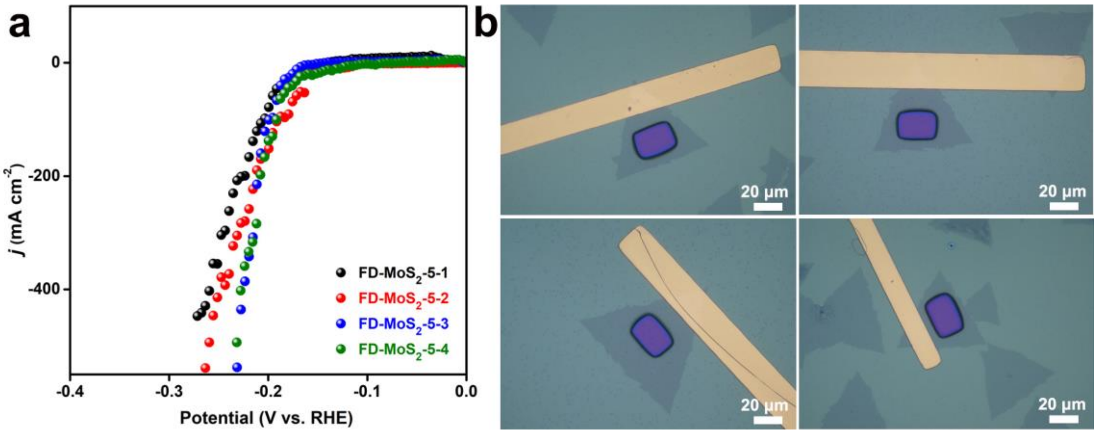


**Supplementary Fig. 21** HER performance of monolayer FD-$MoS_2$-5 catalysts and corresponding optical microscopy images. (a) Polarization curves of different sample areas of monolayer FD-$MoS_2$-5 in 0.5 M $H_2SO_4$. All current values are normalized by the exposed FD-$MoS_2$-5 surface areas. (b) The corresponding optical microscopy images of exposure areas of different FD-$MoS_2$-5 samples. The purplish red part is the exposed FD-$MoS_2$-5 base surface with an area of (20 μm×30 μm) 600 $μm^2$.

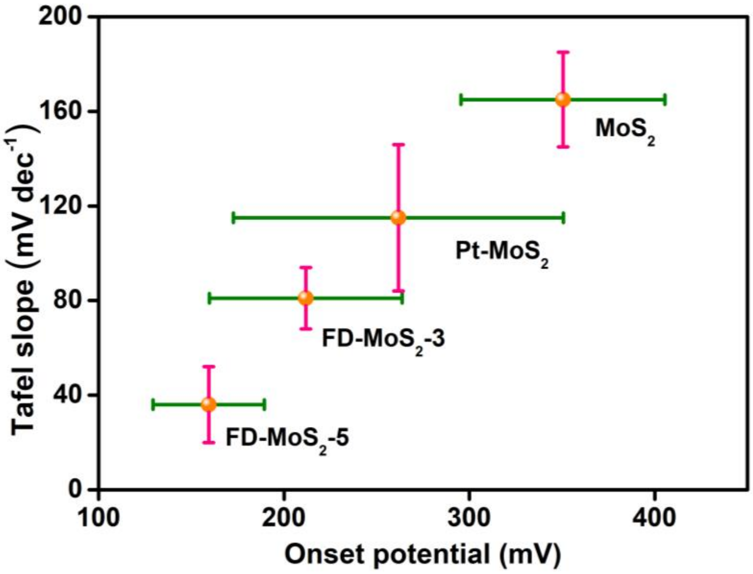


**Supplementary Fig. 22** Comparison of HER performance of pristine $MoS_2$, Pt-$MoS_2$, FD-$MoS_2$-3, and FD-$MoS_2$-5 catalysts. Tafel slopes are plotted corresponding to the onset potentials based on statistical analysis. The polarization curves of these materials are shown in Supplementary Figs. 18–21.

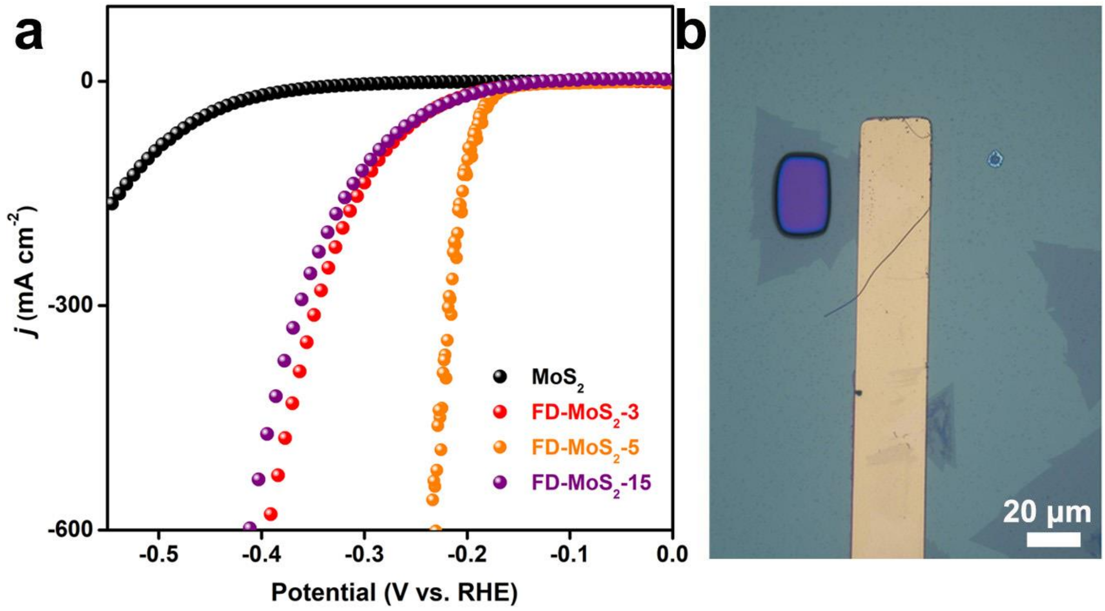


**Supplementary Fig. 23** HER performance of monolayer $MoS_2$, FD-$MoS_2$-3, FD-$MoS_2$-5 and FD-$MoS_2$-15 catalysts and corresponding optical microscopy image of FD-$MoS_2$-15. (a) Polarization curves of monolayer $MoS_2$, FD-$MoS_2$-3, FD-$MoS_2$-5 and FD-$MoS_2$-15 in 0.5 M $H_2SO_4$. All current values are normalized by the exposed surface areas. (b) The purplish red part is the exposed FD-$MoS_2$-15 base surface with an area of (20 μm×30 μm) 600 μm$^2$. Compared with the overpotential of FD-$MoS_2$-5 at 10 mA cm$^{-2}$ of 164 mV, the overpotential of FD-$MoS_2$-15 is 176 mV. This shows that the HER performance of FD-$MoS_2$-15 is inferior to that of FD-$MoS_2$-5.

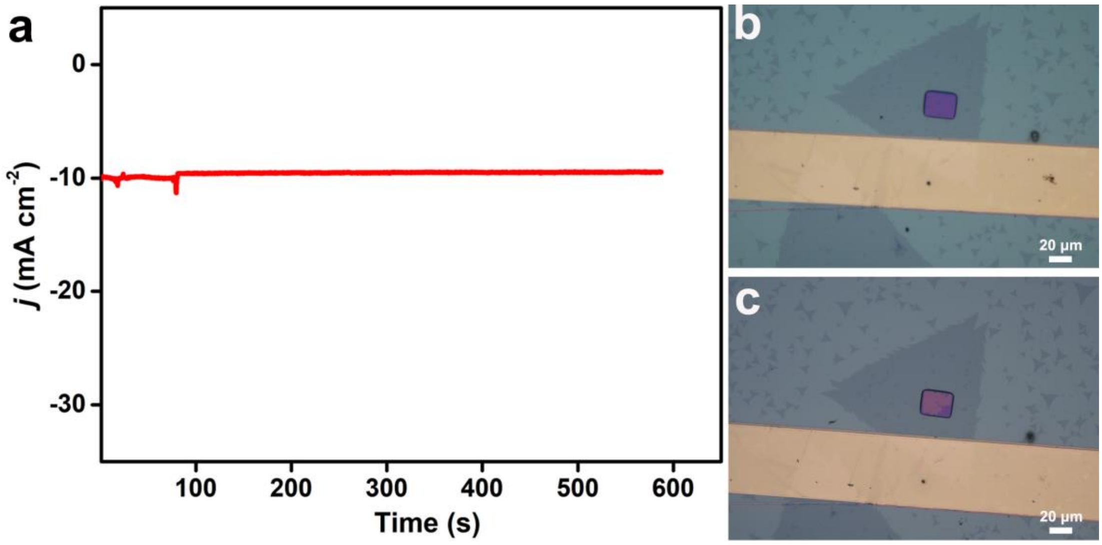


**Supplementary Fig. 24** Electrochemical stability test results of monolayer FD-$MoS_2$-3 and corresponding optical microscopy images. (a) The chronoamperometry results of monolayer FD-$MoS_2$-3 at 10 mA $cm^{-2}$. The optical images of the exposure window before testing (b) and after stability testing (c). The results confirmed that the exposed monolayer FD-$MoS_2$-3 2D material decomposed and extincted after approximately 10 minutes of stability testing.

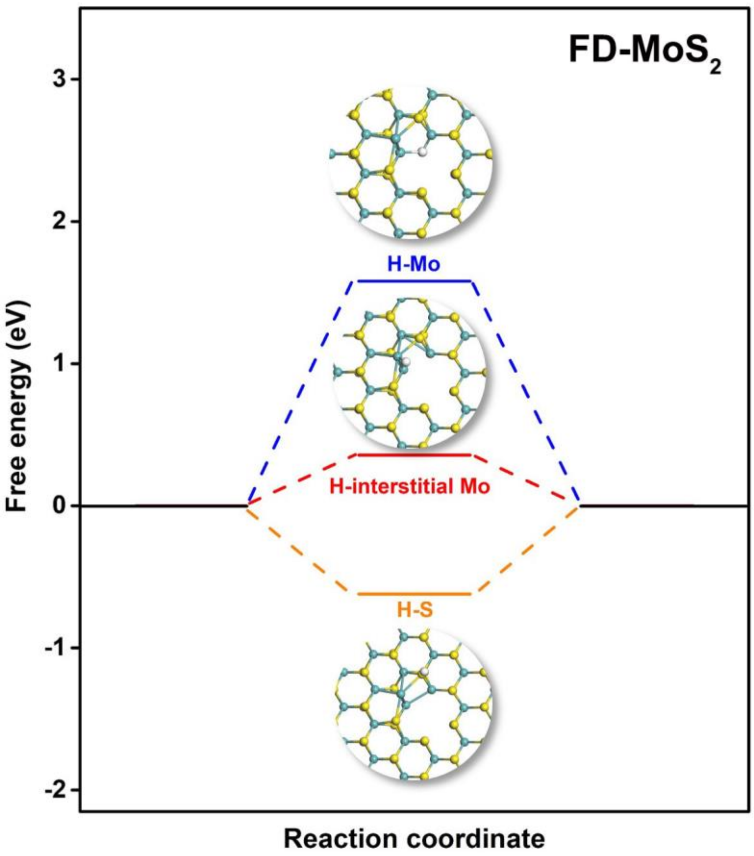


**Supplementary Fig. 25** Free energy diagrams of HER for FD-$MoS_2$ with different active sites. The illustration is the related structure of adsorbed H*. The calculation results show that the free energy of H* adsorption of interstitial Mo atom (in Frankel defects), Mo atom, and S atom are 0.36 eV, 1.58 eV and -0.62 eV, respectively. The results indicate that interstitial Mo atom exhibits the best HER activity compared to other potential active sites.

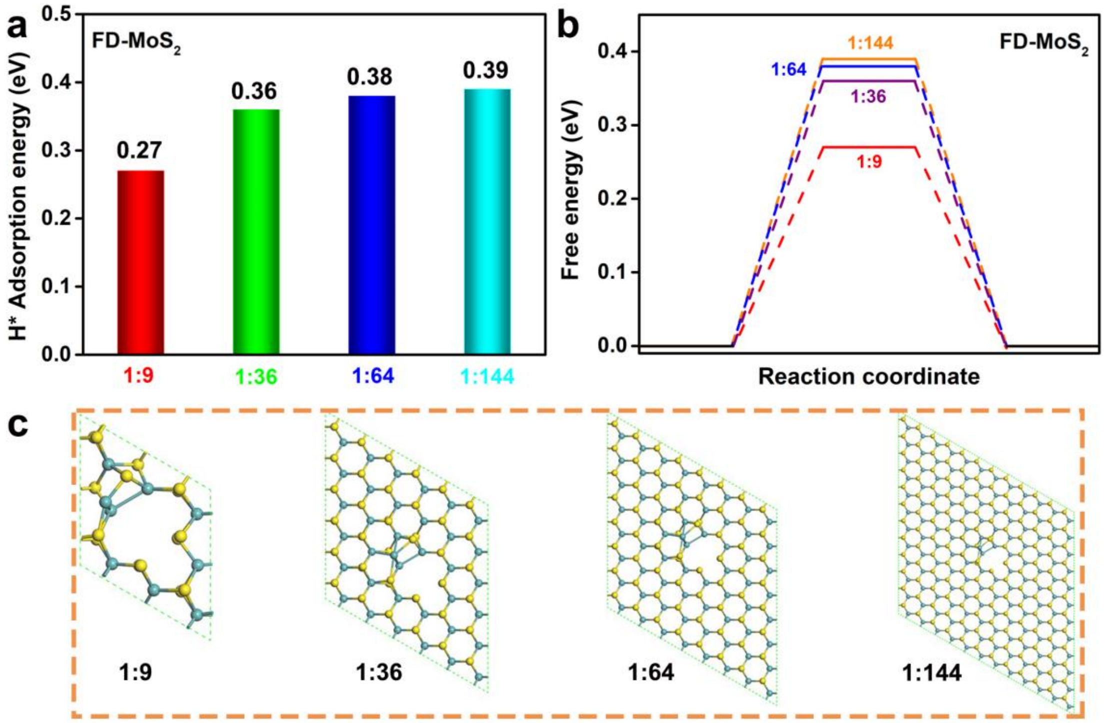


**Supplementary Fig. 26** DFT calculation showing the influence of different concentrations of Frankel defects on the catalytic HER activity. (a,b) The adsorption free energy of H* with different concentrations (1:9, 1:36, 1:64, and 1:144) of Frenkel defects. (c) The atomic model corresponding to different concentrations of Frankel defects. It shows a promotion of HER activity with larger Frenkel defects concentration (0.27 eV of limiting energy on 1:9 compared to 0.36 eV on 1:36). This also indicates that a higher concentration of Frenkel defects in FD-$MoS_2$ will further optimize its HER catalysis performance.

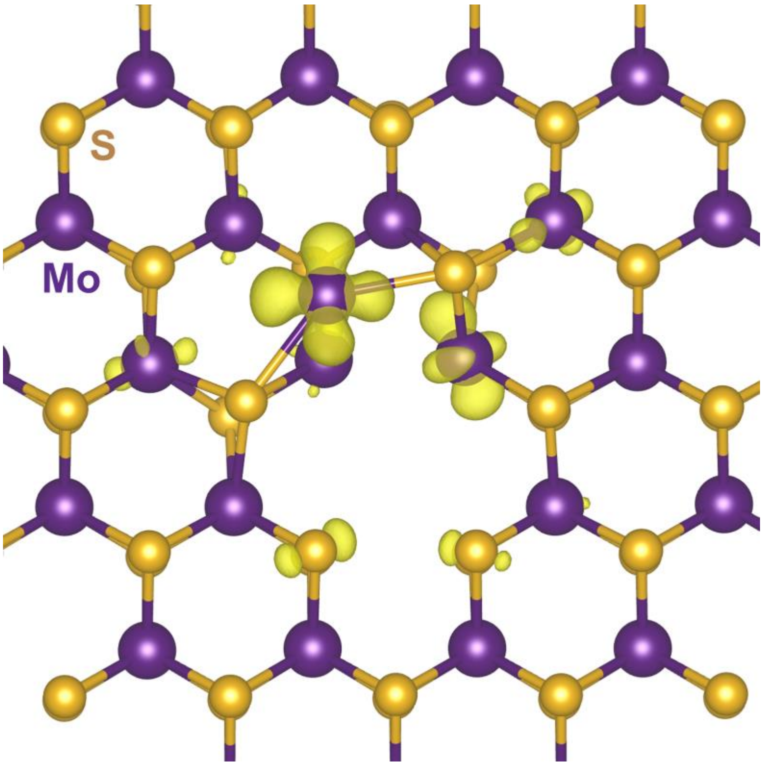


**Supplementary Fig. 27** The partial charge density distribution of the bottom conduction bands for FD-$MoS_2$ atomic structure. The results show that most of the bottom conduction band electrons were distributed on the interstitial Mo and another dangling Mo atom.
